# Nanoassembly-enabled aqueous solid-phase peptide synthesis (ASPPS): a practical DMF-free approach based on the Fmoc strategy

**DOI:** 10.1039/d6ra00715e

**Published:** 2026-04-07

**Authors:** Keiko Hojo, Ayumi Nonaka, Yuki Manabe, Cédric Rentier, Amit Mehrotra, Kazuhito Hioki, Munetaka Kunishima

**Affiliations:** a Faculty of Pharmaceutical Sciences, Kobe Gakuin University Kobe 650-8586 Japan hojo@pharm.kobegakuin.ac.jp kunisima@pharm.kobegakuin.ac.jp; b Cooperative Research Center of Life Sciences, Kobe Gakuin University Kobe 650-8586 Japan; c Biotage Japan Ltd. Tokyo 136-0071 Japan; d Biotage Sweden AB Uppsala 753 18 Sweden

## Abstract

The urgent need for sustainable peptide manufacturing has accelerated efforts to replace conventional *N*,*N*-dimethylformamide (DMF)-based solid-phase peptide synthesis (SPPS) with greener alternatives. Here, we present a practical SPPS protocol that uses water as the reaction medium and eliminates hazardous organic solvents. The method leverages nanoassemblies formed from Fmoc-protected amino acids, coupling reagents, and bases, which create reactive interfacial microenvironments that enhance local concentration and promote efficient peptide bond formation under aqueous conditions. These nanoassemblies are readily prepared without specialized equipment and are compatible with microwave-assisted coupling, enabling scalability and semi-automation using existing SPPS platforms. Using this approach, we synthesized various peptides, including β-endorphin (31 residues), with yields and purities comparable to those obtained using conventional DMF-based SPPS. By integrating DMF-free chemistry with nanoassembly-driven reactivity, this work introduces a reaction-field-based strategy for peptide synthesis and provides a simple and eco-friendly platform aligned with the principles of green chemistry.

## Abstract 2

A nanoassembly-based ASPPS is introduced, where Fmoc-amino acids form non-molecularly dissolved domains in water. These interfacial microenvironments enhance reactivity, enabling rapid and practical peptide synthesis under aqueous coupling conditions.

## Graphical abstract

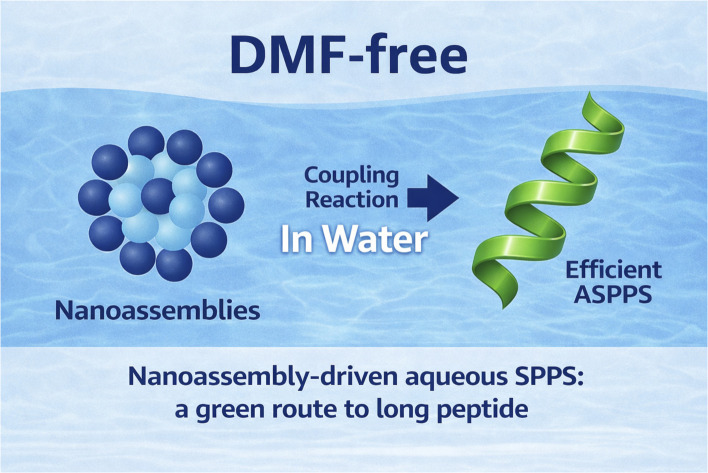

## 1. Introduction

Regulatory restrictions on the use and disposal of organic solvents have become increasingly stringent in recent years, driven by growing environmental and health concerns. Within the framework of green and sustainable chemistry,^1^ the development of safer, non-hazardous alternatives to conventional solvents is urgently needed.^2^ Peptides have attracted considerable attention across diverse fields, particularly in pharmaceutical development,^3–5^ where their chemical synthesis is well established. However, conventional peptide synthesis methods were not designed with environmental sustainability in mind and continue to rely heavily on *N*,*N*-dimethylformamide (DMF) as a solvent. DMF is known to cause soil contamination and was classified as a hazardous substance under the EU's REACH regulation in 2021, leading to drastic restrictions and exposure limits for DMF in the EU, as of December 2023.^6^

Against this backdrop, there is a strong demand for environmentally friendly peptide synthesis technologies that eliminate the use of DMF. This has spurred increased efforts to develop water-based peptide synthesis methods. However, none of the reported water-based approaches have achieved broad applicability, primarily due to limitations in efficiency and compatibility with standard amino acid derivatives.

There are several methods of chemical peptide synthesis, including liquid-phase synthesis, solid-phase synthesis and tagged liquid-phase synthesis. Among these, solid-phase peptide synthesis (SPPS)^7^ is the most widely used, owing to its simplicity, ease of automation, and suitability for diverse peptide sequences.^8,9^ Therefore, we focused our efforts on developing an environmentally friendly SPPS protocol using water, aiming to provide a practical and sustainable alternative to conventional DMF-based methods. However, efficient peptide synthesis in aqueous SPPS (ASPPS) is challenging due to the poor water solubility of commonly used protected amino acids, such as Fmoc-amino acids.^10^ To overcome this limitation, water-soluble protected amino acids have been developed and applied in aqueous synthesis.^11–18^ Unfortunately, these derivatives often suffer from low stability and slow reaction rates, which restrict the range of peptides that can be synthesized and result in low product crude purity.

Although Fmoc-protected amino acids are generally considered unsuitable for aqueous reactions due to their poor solubility, recent studies have begun to explore their use in water-based systems. Several peptide chemists have focused on developing new methodologies for aqueous peptide synthesis, often by dissolving Fmoc-amino acids in water using organic co-solvents or surfactants, as reported by Albericio and co-workers.^19^ While these methods enable aqueous peptide synthesis, they still require significant amounts of organic additives and exhibit slow reaction kinetics, limiting their environmental benefits.

To overcome these challenges, we adopted a different strategy: instead of dissolving protected amino acids in water, we leveraged their inherent insolubility. Previously, we proposed the use of water-dispersible nanoparticles composed of Fmoc-protected amino acids for ASPPS.^20–24^ This approach enabled efficient peptide synthesis under aqueous conditions while maintaining compatibility with the Fmoc strategy. Notably, we observed a unique reaction acceleration phenomenon: when protected amino acids were used as nanoparticles without dissolving in water, the reaction proceeded more efficiently than with water-soluble derivatives. Additionally, this method suppressed racemization during coupling. Despite these advantages, the nanoparticle-based method has limitations that hinder its general applicability. The preparation of nanoparticles requires wet grinding using a planetary ball mill, and the resulting particles are relatively large (∼300 nm), leading to transient dispersion and eventual aggregation in water. These characteristics make the method unsuitable for automation and large-scale synthesis.

Here, we report an ASPPS method that utilizes nanoassemblies formed by mixing water-insoluble Fmoc-protected amino acids with water-soluble coupling reagents and bases. These nanoassemblies provide reactive interfacial microenvironments, exhibiting a reaction-acceleration effect comparable to nanoparticle-mediated systems, and enabling rapid and efficient peptide synthesis without hazardous DMF or energy-intensive milling. We demonstrate applicability across various peptides, including β-endorphin (31 residues). Recent studies^25^ have demonstrated ASPPS *via* salt-based solubilization of Fmoc building blocks; in contrast, our approach operates in a nanoassembled, non-molecularly dissolved state (supported by Tyndall effect and DLS analysis; see SI), which constitutes a fundamentally different reaction environment. This interfacial nanoassembly is critical for maintaining both high reactivity and automation robustness, including line compatibility in flow or microwave-assisted systems. A concise comparison is provided in Table S1. While we avoid assigning a single definitive mechanism, the observed behavior is consistent with on-water-like interfacial reactivity. Overall, this nanoassembly-based approach achieves high efficiency while eliminating hazardous DMF and avoiding specialized equipment or additional additives, offering a scalable and sustainable platform for next-generation peptide synthesis in line with green chemistry principles.

## 2. Experimental

### 2.1 Material and methods

Fmoc-amino acids were obtained from Watanabe Chemical Industries, Ltd. (Hiroshima, Japan). Reagents and solvents were obtained from Tokyo Chemical Industry Co., Ltd. (Tokyo, Japan). Particle size was determined by dynamic light scattering (DLS) using a Zetasizer Nano ZSP (Malvern Panalytical Ltd., Malvern, U.K.). Microwave (MW) reactions were performed using a Biotage® Initiator + SP Wave system (Biotage AB, Uppsala, Sweden). Reversed-phase HPLC was carried out on a Waters Alliance e2695 system (Waters Corp., Milford, MA, U.S.A.) equipped with a COSMOSIL 5C18-AR-II column (Nacalai Tesque Inc., Kyoto, Japan) using a gradient of acetonitrile/water containing 0.1% trifluoroacetic acid (TFA). Mass spectra were recorded on an electrospray ionization quadrupole time-of-flight mass spectrometer (ESI-Q-TOF-MS), micrOTOF-Q (Bruker Daltonik GmbH, Bremen, Germany). Detailed HPLC analysis, integration criteria, and ESI-MS acquisition protocols are provided in the Supporting Information.

### 2.2 General procedure for preparation of nanoassemblies of Fmoc-amino acids containing coupling reagents and bases

Nanoassemblies of Fmoc-Phe-OH prepared with 4-(4,6-dimethoxy-1,3,5-triazin-2-yl)-4-methylmorpholinium chloride (DMT-MM)^26,27^ and NMM (Example): Fmoc-Phe-OH (39 mg, 100 µmol) and 4-methylmorpholine (NMM) (22 µl, 200 µmol) were mixed in a small amount of water using a vortex mixer. After dilution with 5 mL of water, 1 mL of an aqueous solution of DMT-MM (28 mg, 100 µmol) was added and mixed vigorously using a vortex mixer to form aqueous nanoassemblies. Particle size (mean diameter): 12.8 ± 3.24 nm.

### 2.3 MW assisted in-water coupling reaction study using nanoassemblies of Fmoc-amino acids with coupling reagents and bases

H-Gly-Rink amide-TentaGel resin^28^ (46 mg, Gly-content, 25 µmol) was swelled with water, then MW assisted coupling reactions were performed using an Initiator + SP Wave system employing several types of aqueous nanoassemblies of Fmoc-Phe-OH (100 µmol) combined with a coupling reagent and a base. DMT-MM, 1-[bis(dimethylamino)methylene]-1H-benzotriazolium 3-oxide tetrafluoroborate (TBTU),^29,30^ 1-[bis(dimethylamino)methylene]-1H-1,2,3-triazolo[4,5-*b*]pyridinium 3-oxide tetrafluoroborate (TATU),^29,31,32^ O-(6-chlorobenzotriazol-1-yl)-*N*,*N*,*N′*,*N′*-tetramethyluronium tetrafluoroborate (TCTU)^33^ or 1-[bis(dimethylamino)methylene]-1H-benzotriazolium 3-oxide hexafluorophosphate (HBTU)^29,34,35^ were evaluated as the coupling reagent (see SI Section 2). *N*,*N*-diisopropylethylamine (DIEA) or NMM were used for the base. Each reaction mixture was heated to 75 °C by microwave irradiation and kept for 1–10 min. After MW irradiation, the resins were washed with water and 2-propanol for 3 times. Each resin was checked using the ninhydrin (Kaiser) test. These test results are summarized in Table 1.

**Table 1:** Aqueous coupling study using nanoassemblies of Fmoc-amino acids with coupling reagents

Entry	Coupling reagent	Base	MW temp (°C)	Time (min)	Ninhydrin test
1	DMT-MM	NMM	75	10	—
2	DMT-MM	NMM	75	5	—
3	DMT-MM	NMM	75	3	Slightly+
4	TBTU	DIEA	75	10	—
5	TBTU	DIEA	75	5	—
6	TBTU	DIEA	75	3	—
7	TBTU	DIEA	75	1	Slightly+
8	TATU	DIEA	75	5	—
9	TATU	DIEA	75	3	—
10	TATU	DIEA	75	1	+
11	TCTU	DIEA	75	5	—
12	TCTU	DIEA	75	3	—
13	TCTU	DIEA	75	1	+
14	HBTU	DIEA	75	5	+

### 2.4 General procedure for microwave-assisted in-water solid-phase peptide synthesis

#### 2.4.1 Leu-enkephalin amide

ASPPS was carried out according to the protocol shown in Table 3. H-Leu-Rink amide-TentaGel resin (100 mg, amino group content, 25 µmol) was swollen in water, and aqueous nanoassemblies (6 mL, 16.7 mM with respect to Fmoc-amino acid) prepared from Fmoc-amino acid (100 µmol), TBTU (100 µmol), and DIEA (200 µmol) were coupled sequentially onto the resin. MW-assisted coupling reactions were performed at 75 °C for 10 min using an Initiator^+^ SP Wave system. Fmoc deprotection was carried out with 20% piperidine–ethyl acetate (EtOAc) solution.^24^ After completion of the synthesis, the peptide resin (H-Tyr(*t*Bu)-Gly-Gly-Phe-Leu-PEG-grafted Rink amide resin) was washed with ethanol and dried *in vacuo*. The resin was treated with TFA–triisopropylsilane (TIPS)–water (92 : 4 : 4, 15 mL) for 2 h at room temperature. The resin was removed by filtration, and TFA was evaporated under N_2_ stream. Diethyl ether was added to the residue, yielding a white precipitate of crude peptide. This crude peptide was subjected to HPLC analysis (220 nm). A dominant peak was observed at a retention time of 18.4 min with a calculated purity of 96.0%. ESI-MS (TOF) *m*/*z*: 555.3737 [M + H]^+^ (calcd. for C_28_H_39_N_6_O_6_, 555.2931).

#### 2.4.2 Dermorphin

ASPPS was carried out according to the protocol shown in Table 3. H-Ser(*t*Bu)-Rink amide-TentaGel resin (100 mg, amino group content, 25 µmol) was swollen in water, and aqueous nanoassemblies (6 mL, 16.7 mM with respect to Fmoc-amino acid) prepared from Fmoc-amino acid (100 µmol), TBTU (100 µmol), and DIEA (200 µmol) were coupled sequentially onto the resin. MW-assisted coupling reactions were performed at 75 °C for 10 min using an Initiator^+^ SP Wave system. Fmoc deprotection was carried out with 20% piperidine–EtOAc solution. After completion of the synthesis, the peptide resin (H-Tyr(*t*Bu)- DAla-Phe-Gly-Tyr(*t*Bu)-Pro-Ser(*t*Bu)-Rink amide-TentaGle resin) was washed with ethanol and dried *in vacuo*. The resin was treated with TFA–TIPS–water (92 : 4 : 4, 15 mL) for 2 h at room temperature. The resin was removed by filtration, and TFA was evaporated under N_2_ stream. Diethyl ether was added to the residue, yielding a white precipitate of crude peptide. This crude peptide was subjected to HPLC analysis (220 nm). Two peaks at 17.8 and 18.6 min were observed and assigned to *cis*/*trans* conformers, and their combined purity was calculated to be 97%. ESI-MS (TOF) *m*/*z*: 803.3705 [M + H]^+^ (calcd. for C_40_H_51_N_8_O_10_, 803.3728).

#### 2.4.3 β-Endorphin

ASPPS was carried out according to the protocol shown in Table 3. H-Glu(*t*Bu)-Rink amide-TentaGel resin (100 mg, amino group content, 25 µmol) was swollen in water, and aqueous nanoassemblies (6 mL, 16.7 mM with respect to Fmoc-amino acid) prepared from Fmoc-amino acid (100 µmol), TBTU (100 µmol), and DIEA (200 µmol) were coupled sequentially onto the resin. MW-assisted coupling reactions were performed at 75 °C for 10 min using an Initiator^+^ SP Wave system. Fmoc deprotection was carried out with 20% piperidine–EtOAc solution. After completion of the synthesis, the protected peptide resin was washed with ethanol and dried *in vacuo*. The resin was treated with TFA–TIPS–water–thioanisole–2,2′-(ethylenedioxy)diethanethiol (92 : 2:2 : 2 : 2, 5 mL) for 2 h at room temperature. The resin was removed by filtration, and TFA was evaporated under N_2_ stream. Ether was added to the residue, yielding a white precipitate of crude peptide. This crude peptide was subjected to HPLC analysis (220 nm). The major peak was observed at a retention time of 26.3 min with a calculated purity of 56%. After HPLC purification, 3.2 mg of pure peptide (TFA salt) was obtained (3.1% isolated yield). Due to partial co-elution with impurities, repeated HPLC purification was required, resulting in a reduced isolated yield. ESI-MS (TOF): *m*/*z* 1733.0737 ([M + 2H]^2+^) and *m*/*z* 1155.7189 ([M + 3H]^3+^) which corresponds to C_158_H_253_N_40_O_45_S (calculated monoisotopic mass: 3463.8537).

## 3. Results and discussion

Selecting the most suitable amino acid derivatives is one of the most critical - and occasionally challenging – aspects of peptide synthesis. Currently, Fmoc-protected amino acids are widely used as building blocks in SPPS due to their broad applicability and commercial availability. However, Fmoc-amino acids exhibit poor solubility in water. Meanwhile, micelle-mediated synthesis has gained attention as a promising strategy for aqueous-phase reactions in industrial chemistry.^36^ In most reported cases, surfactants form micellar systems that either provide a reaction interface in aqueous media or enhance the solubility of hydrophobic reactants.^37–39^ Structurally, Fmoc-amino acids consist of a hydrophobic Fmoc group and a hydrophilic carboxyl group, giving them amphiphilic properties similar to those of surfactants. Inspired by this surfactant-like nature, we began exploring peptide synthesis methods that leverage the intrinsic properties of Fmoc-amino acids.

**Fig. 1:**
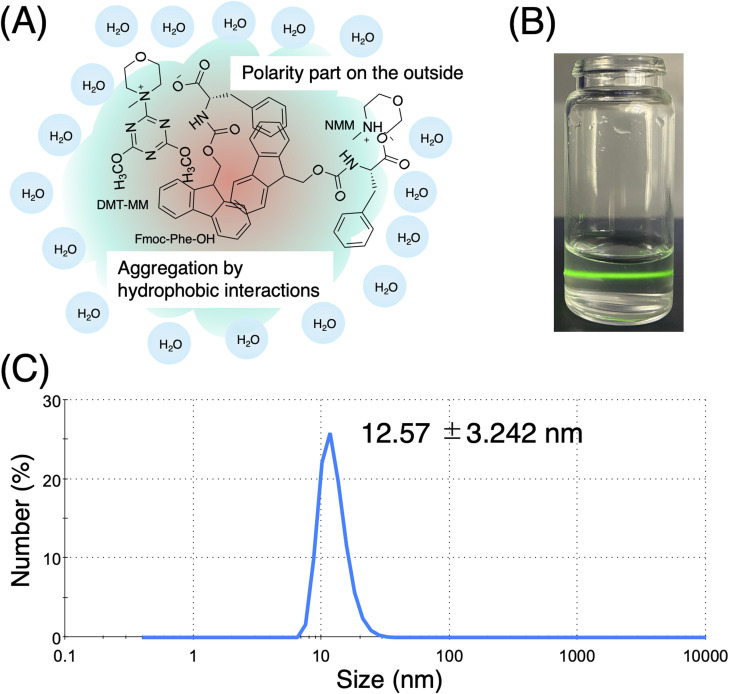
Aqueous nanoassemblies of Fmoc-Phe-OH with DMT-MM and NMM. (A) Schematic diagram of nanoassemblies; (B) photo image of Tyndall effects by nanoassemblies; (C) particle size distribution (number-weighted) for nanoassemblies showing a sharp peak at 12.57 nm (*Z*-average = 17.96 nm; PdI = 0.085).

We discovered that mixing water-insoluble Fmoc-protected amino acids with water-soluble coupling reagents and bases in specific ratios leads to the spontaneous formation of nanoassemblies. For example, powdered Fmoc-Phe-OH was blended with two equivalents of NMM, followed by the addition of an aqueous solution of DMT-MM, resulting in the formation of Fmoc-Phe-OH/DMT-MM nanoassemblies (Fig. 1A). Although the solution appeared clear to the naked eye, the Tyndall effect was observed upon laser irradiation, confirming the presence of colloidal particles (Fig. 1B). DLS analysis revealed that these nanoassemblies had an average diameter of 12.8 ± 3.24 nm (Fig. 1C).

To evaluate the feasibility of using these nanoassemblies as building blocks in aqueous peptide synthesis, we conducted a microwave-assisted solid-phase coupling reaction between the Fmoc-Phe-OH nanoassembly (prepared with DMT-MM and NMM) and H-Gly-Rink amide-TentaGel resin. The reaction temperature was maintained between 75 °C using a semi-automated peptide synthesizer (Biotage® Initiator^+^ SP Wave). Additionally, we prepared nanoassemblies of Fmoc-Phe-OH using various coupling reagents^29^ which are highly active and suitable for use *in situ*—TBTU, TATU, TCTU, and HBTU—in combination with appropriate bases. The nanoassembly composed of Fmoc-Phe-OH, (1-cyano-2-ethoxy-2-oxoethylidenaminooxy)dimethylaminomorpholinocarbenium hexafluorophosphate (COMU)^29,40^ and DIEA could not be prepared because COMU exhibits poor solubility in water. These assemblies were subjected to microwave-assisted coupling reactions, and the results are summarized in Table 1.

All aqueous coupling reactions using nanoassemblies, except those prepared with HBTU, proceeded quantitatively and were completed within 5 minutes. Notably, the nanoassembly formed with TBTU and DIEA exhibited the highest reaction rate, comparable to conventional DMF-based conditions. Also, good results were obtained even when using TATU and TCTU.

The observed acceleration of reactions using nanoassemblies should be attributed to several factors: the local concentration effect, where reactants are confined within microenvironments, the preorientation effect, in which reactive sites are brought into close proximity through hydrophobic and electrostatic interactions, and the dehydration of polar groups, such as carboxyl and amino groups in a hydrophobic environment, which facilitates peptide bond formation. These effects collectively may account for the enhanced reaction efficiency observed in aqueous nanoassembly-based systems. The acceleration observed in our coupling reactions is likely related to the “on-water” mechanism, where water-insoluble substrates react more rapidly at the water–organic interface.^41,42^ As discussed by Cortes-Clerget and colleagues, on-water reactions exploit interfacial hydrogen bonding and hydrophobic interactions to stabilize transition states, in contrast to homogeneous in-water systems where substrates are fully solvated.^43,44^ Similarly, the group led by Kunishima demonstrated that micellar interfaces dramatically accelerate bimolecular coupling reactions - up to 2000-fold - through local concentration and preorientational effects.^45,46^ While our system does not rely on conventional surfactant-based micelles, the nanoassemblies described here provide analogous hydrophobic microdomains that maintain reactants in a partially dehydrated and spatially localized state. This interfacial nanoassembly environment is therefore considered a key factor underlying the markedly enhanced reaction rates compared to conventional aqueous systems employing fully dissolved, water-soluble protected amino acids (Table 2).^16,21^

**Table 2:** Comparative study of coupling reactions using the DMT-MM/NMM method at room temperature: nanoassemblies of Fmoc-amino acids *versus* water-soluble protected amino acids.^19,21^

Entry	Component	Solvent	Time (min)	Coupling yield (%)	Ninhydrin test
1	Nanoassemblies of Fmoc-Phe-OH	Water	15	Quant.	—
2	Water-dispersible nanoparticles of Fmoc-Phe-OH	Water	15	Quant.	—
3	Sps-Phe-OH^a^	Water (2.0% Triton aq.)	60	ND	+
4	Sps-Phe-OH	Water (2.0% Triton aq.)	180	32	+
5	Sps-Phe-OH	50% ethanol aq.	60	47	+
6	Sps-Phe-OH	50% ethanol aq.	180	Quant.	—

a Sps: 2-(4-sulfophenylsulfonyl)ethoxy carbonyl; categorized as a water-soluble and base-lability protecting group for amino acids.

**Fig. 2:**
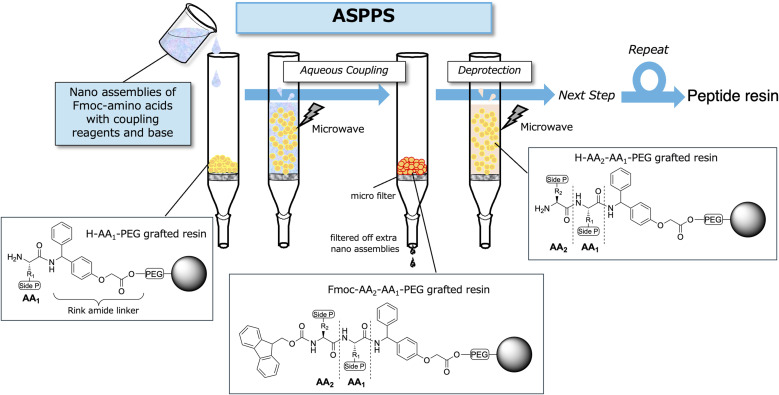
ASPPS using aqueous nanoassemblies of Fmoc-amino acids.

**Fig. 3:**
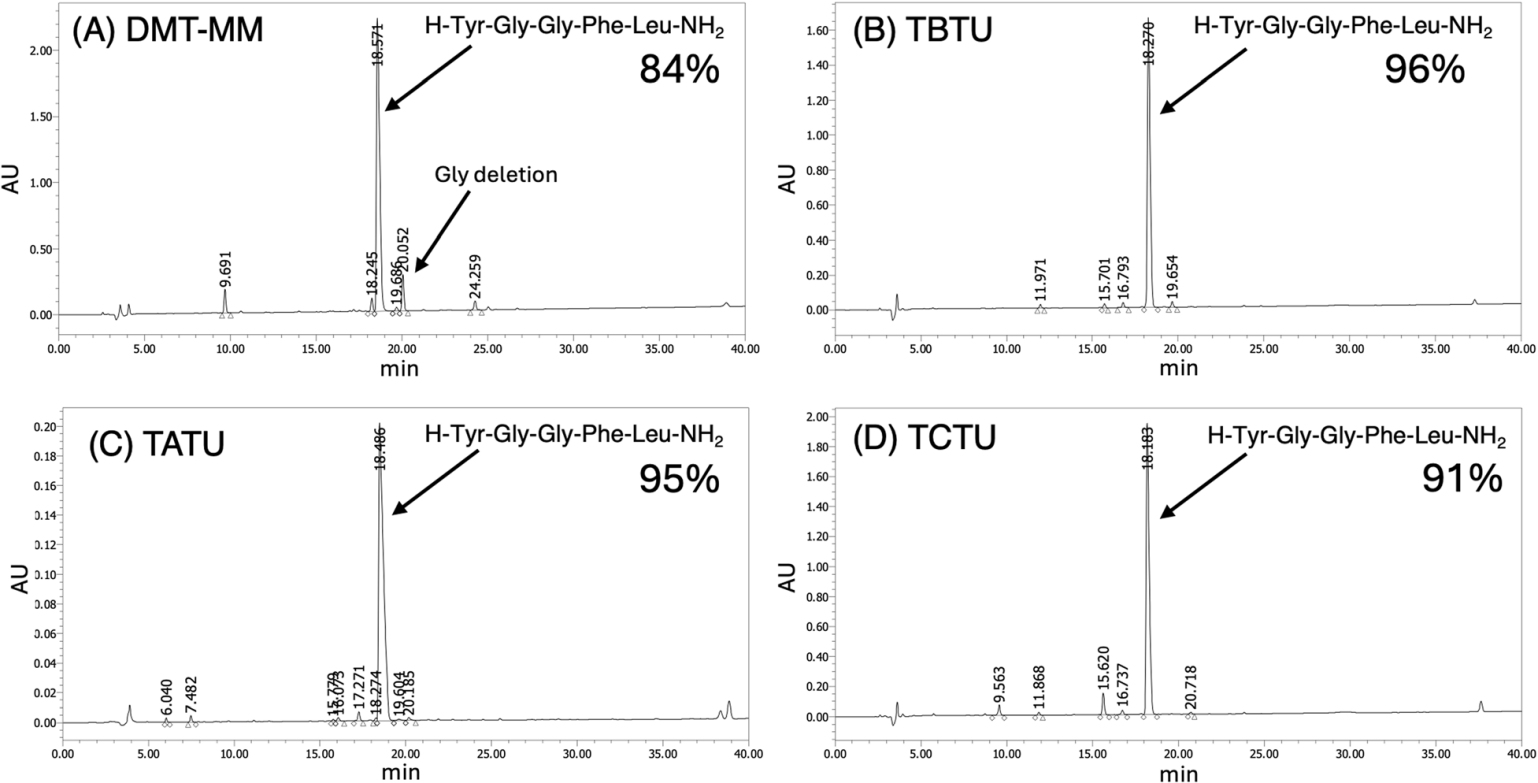
HPLC profiles of crude Leu-enkephalin amide by ASPPS using nanoassemblies Fmoc-amino acids with (A) DMT-MM and NMM; (B) TBTU and DIEA; (C) TATU and DIEA; (D) TCTU and DIEA. Elution was carried out over 40 min at a flow rate of 1 mL min^−1^ with a linear gradient from 90 : 10 to 50 : 50 mixture of 0.1% aqueous TFA and 0.1% TFA in acetonitrile.

Next, we performed ASPPS of a short-chain model peptide, Leu-enkephalin amide (H-Tyr-Gly-Gly-Phe-Leu-NH_2_),^47^ to evaluate the effectiveness of the nanoassembly-based method (Fig. 2). For the aqueous coupling reactions, several types of nanoassemblies were prepared using water-soluble coupling reagents, including DMT-MM, TBTU, TATU, and TCTU. The model peptide was synthesized according to the aqueous synthesis protocol summarized in Table 3. Pre-loaded resins were employed to decouple sequence-dependent variables from the evaluation of nanoassembly-mediated coupling efficiency (see SI Section 3). Coupling reactions were conducted under microwave irradiation at 75 °C for 10 min. Fmoc deprotection was carried out using 20% piperidine in EtOAc for 20 min at room temperature. EtOAc is considered a green solvent, as it readily hydrolyzes into acetic acid and ethanol in the environment.^48,49^ Thus, this protocol eliminates the use of DMF entirely, relying only on water and green solvents, making it more environmentally friendly. However, piperidine is still required in the deprotection step. The nanoassemblies of Fmoc-amino acids with coupling reagents and bases were sequentially coupled onto H-Leu-Rink amide-TentaGel resin. Upon completion of the SPPS cycle, the peptide was cleaved from the resin using TFA. The HPLC analysis results for H-Tyr-Gly-Gly-Phe-Leu-NH_2_ are shown in Fig. 3. Chromatographic integration was performed using a uniform protocol (see SI Section 5-1).

**Table 3:** The protocols for ASPPS using nanoassemblies of Fmoc amino acids

Step	Reagents	Volume^a^ (mL)	Temp (°C)	Time
Wash	Water	5	25	3 min × 5
Coupling reaction	Nanoassemblies of Fmoc-amino acids with coupling agent and base	6	75	10 min
Wash	Water	5	25	3 min × 5
Wash	EtOAc	5	25	3 min × 3
Deprotection	20% pip-EtOAc	5	25	20 min
Wash	EtOAc	5	25	3 min × 3

a The solvent volumes per 100 mg resin.

**Fig. 4:**
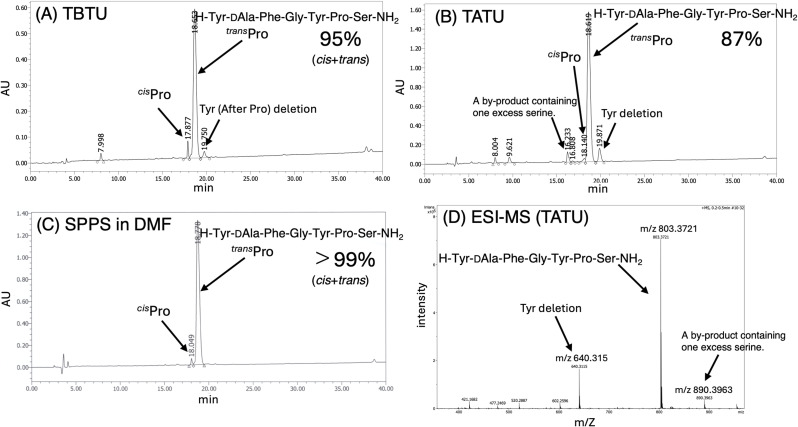
(A) HPLC profiles of crude dermorphin by ASPPS using nanoassemblies Fmoc-amino acids with TBTU and DIEA. Elution was carried out over 40 min at a flow rate of 1 mL min^−1^ with a linear gradient from 90 : 10 to 50 : 50 mixture of 0.1% aqueous TFA and 0.1% TFA in acetonitrile.; (B) HPLC profiles of crude dermorphin by ASPPS using nanoassemblies Fmoc-amino acids with TATU and DIEA; (C) HPLC profiles of crude dermorphin by conventional SPPS using DIC/HOBt method in DMF. (D) ESI-MS (TOF) spectra of crude dermorphin by ASPPS using nanoassemblies Fmoc-amino acids with TATU and DIEA. The observed mass was *m*/*z* 803.3705 ([M + H]^+^), which corresponds to C_40_H_51_N_8_O_10_ (calculated: 803.3728).

In the case of the nanoassembly formed with TBTU, the chromatogram of the crude peptide displayed a single sharp peak (Fig. 3B), indicating excellent purity—comparable to or even exceeding that of conventional SPPS in DMF. Similarly, using nanoassemblies formed with TATU and TCTU also yielded crude peptides with single, well-defined peaks (Fig. 3C and D). In Fig. 3A, using DMT-MM-based nanoassembly produced a chromatogram with a dominant peak corresponding to the target peptide, accompanied by minor byproduct peaks in the latter part of the retention time. The peak at 20.05 min is consistent with YGFL, arising from a glycine-deleted sequence. If the activated form of the protected amino acid becomes partially dissolved in water, hydrolysis may become more pronounced. The relatively lower reactivity observed for Gly, which lacks a hydrophobic side chain, is consistent with the hypothesis that the reaction proceeds primarily within hydrophobic interfacial microdomains, although we refrain from further mechanistic interpretation at this stage. Importantly, no significant side reactions were detected during microwave-assisted SPPS using our environmentally friendly nanoassembly-based protocol. Furthermore, nanoassemblies formed with uronium-type coupling reagents enabled high-purity peptide synthesis in aqueous conditions. We also synthesized a 7-residue neuropeptide, dermorphin amide (H-Tyr-DAla-Phe-Gly-Tyr-Pro-Ser-NH_2_)^50^ using nanoassemblies of Fmoc-amino acids formed with TBTU and TATU. In both cases, the HPLC profiles of the crude peptides showed a dominant peak with only minor impurities and no significant detectable by-products or deletion sequences (Fig. 4A and B).

**Fig. 5:**
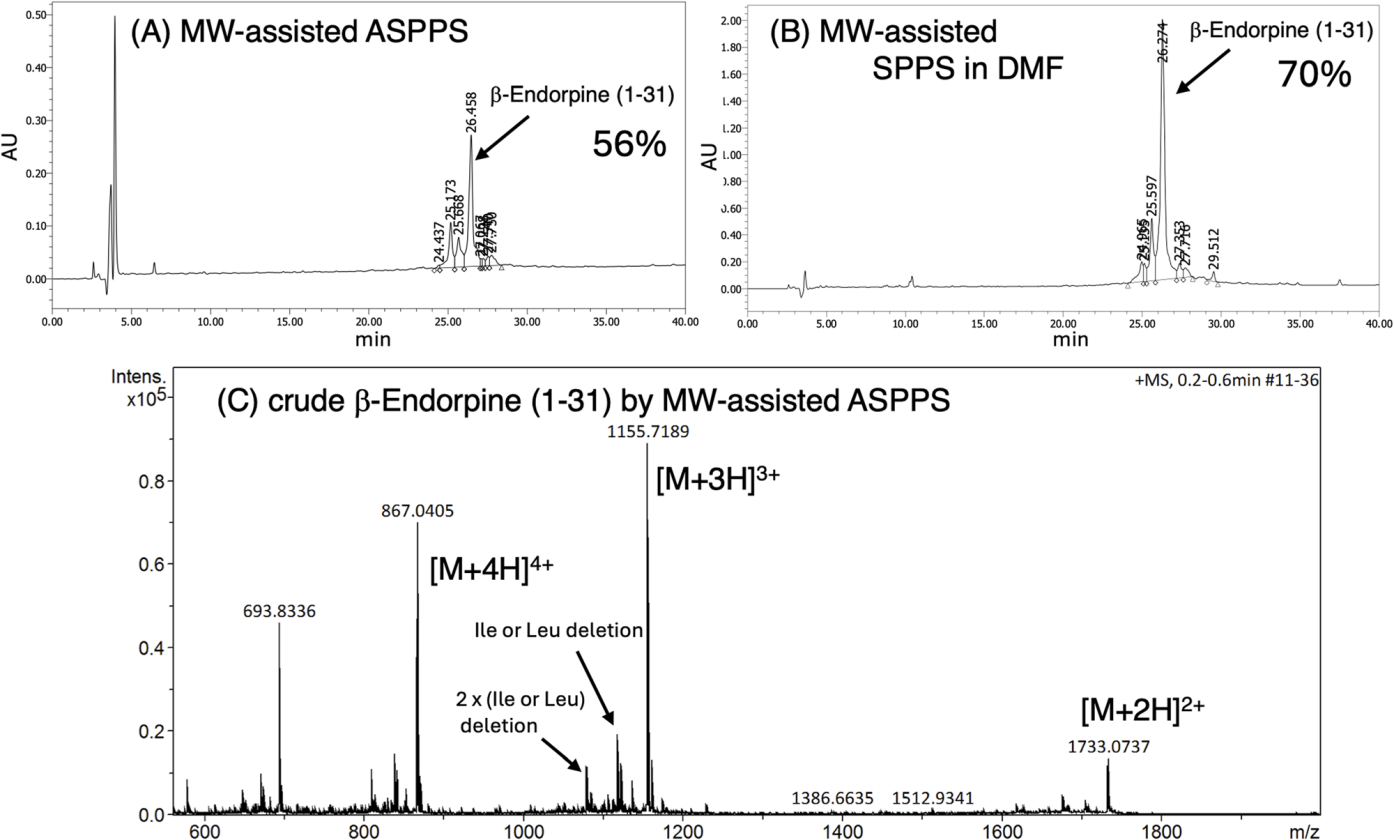
HPLC profiles of crude β-endorphin (1–31) (A) obtained by ASPPS employing nanoassemblies. Elution was carried out over 40 min at a flow rate of 1 mL min^−1^ with a linear gradient from 90 : 10 to 50 : 50 mixture of 0.1% aqueous TFA and 0.1% TFA in acetonitrile; (B) obtained by conventional SPPS employing Fmoc strategy in DMF; (C) ESI-MS (TOF) spectra of crude β-endorphin (1–31) obtained by ASPPS. The observed doubly charged species was *m*/*z* 1733.0737 ([M + 2H]^2+^), which corresponds to C_158_H_253_N_40_O_45_S (calculated monoisotopic mass: 3463.8537). In addition, a triply charged species was detected at *m*/*z* 1155.7189 ([M + 3H]^3+^), consistent with the same molecular formula.

Finally, we attempted the synthesis of a 31-residue peptide, β-endorphin (H-Tyr-Gly-Gly-Phe-Met-Thr-Ser-Glu-Lys-Ser-Gln-Thr-Pro-Leu-Val-Thr-Leu-Phe-Lys-Asn-Ala-Ile-Ile-Lys-Asn-Ala-Tyr-Lys-Lys-Gly-Glu-NH_2_),^51^ using the same protocol as described above (Table 3) and nanoassemblies formed with TBTU. All coupling reactions were performed as double couplings for 10 minutes at 75 °C. Although minor deletion peaks were observed, β-endorphin was obtained as the main product (Fig. 5A). The MS spectra (Fig. 5C) suggest that these deletion peaks can be attributed to residues such as Ile and Leu, based on the observed mass differences. Some of these residues follow sterically hindered amino acids and correspond to positions known to be challenging for peptide bond formation. These tendencies are consistent with those observed in conventional SPPS. Supporting MS data are provided in Fig. S32 (SI). The chromatogram shows a dominant peak at 26.4 min with a calculated purity of 56%.

To our knowledge, this represents the first successful reported synthesis of a peptide longer than 30 residues using an aqueous coupling method. These results demonstrate that our nanoassembly-based approach is highly effective and broadly applicable, including for the synthesis of long-chain peptides.

## 4. Conclusion

In this study, we developed a nanoassembly-based approach that utilizes Fmoc-protected amino acids without dissolving them in water as individual molecules, instead forming reactive nanoassemblies with coupling reagents and bases. This strategy leverages the inherent insolubility of protected amino acids to create interfacial reaction fields, which play a key role in accelerating coupling reactions under aqueous conditions. The resulting protocol enables rapid and environmentally friendly SPPS, completely eliminating DMF. Nanoassemblies are easily prepared without specialized equipment and allow efficient microwave-assisted coupling reactions in water. Using this method, we successfully synthesized various peptides, including β-endorphin (31 residues), marking the first demonstration of a peptide exceeding 30 residues synthesized *via* an aqueous coupling strategy. Compared to nanoparticle- or micelle-based approaches, this method avoids the use of surfactants and energy-intensive processes such as wet-milling, and is based on a nanoassembly-driven interfacial reaction environment rather than micellar solubilization, thereby offering advantages in energy efficiency and waste reduction. Importantly, this protocol eliminates hazardous solvents such as DMF and relies only on water and green solvents, aligning with the principles of green chemistry. Although quantitative environmental metrics such as E-factor were not determined in this study, the significant reduction in organic solvent usage strongly indicates improved sustainability compared to conventional SPPS. Future work will include comprehensive green chemistry assessments to further validate these benefits. Overall, the nanoassembly-based approach provides a simple, scalable, and environmentally benign platform for peptide synthesis, with strong potential to advance sustainable manufacturing in peptide chemistry.

## Author contributions

K. Hojo: writing-original draft, conceptualization, investigation, and funding acquisition. A. Nonaka: peptide synthesis and analytical measurement. Y. Manabe: peptide synthesis and analytical measurement. C. Rentier: scientific and technical input, review & editing. A. Mehrotra: scientific and technical input, review & editing. K. Hioki: investigation and review & editing, M. Kunishima: review & editing and supervision.

## Conflicts of interest

C. Rentier is an employee of Biotage Japan Ltd. A. Mehrotra is an employee of Biotage Sweden AB. The authors declare that related patent applications (WO2024-143052 and JP Application No. 2025-149977) exist concerning the aqueous nanoassembly methodology. K. Hojo is named as an inventor on these applications.

## Supplementary Material

RA-016-D6RA00715E-s001

## Data Availability

All data supporting the findings of this study are available within the article and its supplementary information (SI). Supplementary information: particle size of nanoassemblies (DLS analysis data) and peptide characterization (ESI-Q-TOF-MS spectra and analytical HPLC profiles). See DOI: https://doi.org/10.1039/d6ra00715e.
