# Supplementary material for: Nanoassembly-enabled aqueous solid-phase peptide synthesis (ASPPS): a practical DMF-free approach based on the Fmoc strategy
Source: RSC Adv. 2026 Apr 7;16(20):18073–82. doi: 10.1039/d6ra00715e (PMC13055598; doi:10.1039/d6ra00715e)
Supplement: RA-016-D6RA00715E-s001 [file RA-016-D6RA00715E-s001.pdf]

Supplementary Information (SI) for RSC Advances.
This journal is © The Royal Society of Chemistry 2026

Supplementary information

Nanoassembly-Enabled Aqueous Solid-Phase Peptide Synthesis (ASPPS): Practical DMF-Free Approach Based on the Fmoc Strategy

Keiko Hojo*[a,b], Ayumi Nonaka[a], Yuki Manabe[a], Cédric Rentier[c], Amit Mehrotra[d], Kazuhito Hioki[a,b], and Munetaka Kunishima*[a,b]

[a]*Faculty of Pharmaceutical Sciences, Kobe Gakuin University, Kobe 650-8586, Japan. E-mail: hojo@pharm.kobegakuin.ac.jp, kunisima@pharm.kobegakuin.ac.jp*

[b]*Cooperative Research Center of Life Sciences, Kobe Gakuin University, Kobe 650-8586, Japan.*

[c]*Biotage Japan Ltd., Tokyo 136-0071, Japan.*

[d]*Biotage Sweden AB, Uppsala 753 18, Sweden.*

Table of contents

1. Side-by-side comparison of ASPPS approaches

Table S1. Side-by-side comparison of ASPPS approaches: nanoassembly-enabled ASPPS (this work) versus salt-based molecular solubilization (e.g., Wellings et al., 2026). -------------------- 5

2. Coupling reagents

2-1. Structure of coupling reagents used in this study -- 7

3. DLS analysis of nanoassemblies

3-1. DLS Measurement and Data Interpretation -- 8

3-1-1. Measurement and Analysis --- 8

3-1-2. Decision Rule for Mode Diameter --- 8

3-1-3. Representative Examples --8

Figure S1. (a) Particle size distribution (number-weighted) for Fmoc-DAla-OH with TBTU and DIEA; (b) Particle size distribution (intensity-weighted); (c) Particle size distribution (intensity-weighted) after 5 min ultrasonication. -- 9

3-2. Particle size distribution for nanoassemblies ---10

3-2-1. Particle size distribution for nanoassemblies of Fmoc-amino acids with DMT-MM and NMM

Figure S2. (a) Particle size distribution (number-weighted) for nanoassemblies of Fmoc-Gly-OH with DMT-MM and NMM; (b) Particle size distribution (intensity-weighted) -------------------10

Figure S3. (a) Particle size distribution (number-weighted) for nanoassemblies of Fmoc-Phe-OH

with DMT-MM and NMM; (b) Particle size distribution (intensity-weighted) -------------------10
Figure S4. (a) Particle size distribution (number-weighted) for nanoassemblies of Fmoc-Tyr(tBu)-OH with DMT-MM and NMM; (b) Particle size distribution (intensity-weighted) --------------11
3-2-2. Particle size distribution for nanoassemblies of Fmoc-amino acids with TBTU and DIEA-- 11
Figure S5. (a) Particle size distribution (number-weighted) for nanoassemblies of Fmoc-Gly-OH with TBTU and DIEA; (b) Particle size distribution (intensity-weighted) ------------------------ 11
Figure S6. (a) Particle size distribution (number-weighted) for nanoassemblies of Fmoc-Phe-OH with TBTU and DIEA; (b) Particle size distribution (intensity-weighted) ------------------------- 12
Figure S7. (a) Particle size distribution (number-weighted) for nanoassemblies of Fmoc-Tyr(tBu)-OH with TBTU and DIEA; (b) Particle size distribution (intensity-weighted) -------------------- 12
Figure S8. (a) Particle size distribution (number-weighted) for nanoassemblies of Fmoc-Ala-OH with TBTU and DIEA; (b) Particle size distribution (intensity-weighted) ------------------------- 13
Figure S9. (a) Particle size distribution by number of nanoassemblies of Fmoc-Asn(Trt)-OH with TBTU and DIEA; (b) Particle size distribution by scattering intensity ----------------------------- 13
Figure S10. (a) Particle size distribution by number of nanoassemblies of Fmoc-Asp(OtBu)-OH with TBTU and DIEA; (b) Particle size distribution by scattering intensity ---------------------- 14
Figure S11. (a) Particle size distribution by number of nanoassemblies of Fmoc-Arg(Pbf)-OH with TBTU and DIEA; (b) Particle size distribution by scattering intensity ---------------------------- 14
Figure S12. (a) Particle size distribution by number of nanoassemblies of Fmoc-Cys(Trt)-OH with TBTU and DIEA; (b) Particle size distribution by scattering intensity ---------------------------- 15
Figure S13. (a) Particle size distribution by number of nanoassemblies of Fmoc-Gln(Trt)-OH with TBTU and DIEA; (b) Particle size distribution by scattering intensity ---------------------------- 15
Figure S14. (a) Particle size distribution by number of nanoassemblies of Fmoc-Glu(OtBu)-OH with TBTU and DIEA; (b) Particle size distribution by scattering intensity ---------------------- 16
Figure S15. (a) Particle size distribution by number of nanoassemblies of Fmoc-His(Boc)-OH with TBTU and DIEA; (b) Particle size distribution by scattering intensity ---------------------------- 16
Figure S16. (a) Particle size distribution by number of nanoassemblies of Fmoc-Ile-OH with TBTU and DIEA; (b) Particle size distribution by scattering intensity ---------------------------- 17
Figure S17. (a) Particle size distribution by number of nanoassemblies of Fmoc-Leu-OH with TBTU and DIEA; (b) Particle size distribution by scattering intensity ---------------------------- 17
Figure S18. (a) Particle size distribution by number of nanoassemblies of Fmoc-Lys(Boc)-OH with TBTU and DIEA; (b) Particle size distribution by scattering intensity ----------------------------

18

Figure S19. (a) Particle size distribution by number of nanoassemblies of Fmoc-Met-OH with TBTU and DIEA; (b) Particle size distribution by scattering intensity ---------------------------- 18

Figure S20. (a) Particle size distribution by number of nanoassemblies of Fmoc-Pro-OH with TBTU and DIEA; (b) Particle size distribution by scattering intensity ---------------------------- 19

Figure S21. (a) Particle size distribution by number of nanoassemblies of Fmoc-Ser(tBu)-OH with TBTU and DIEA; (b) Particle size distribution by scattering intensity ---------------------------- 19

Figure S22. (a) Particle size distribution by number of nanoassemblies of Fmoc-Thr(tBu)-OH with TBTU and DIEA; (b) Particle size distribution by scattering intensity ---------------------------- 20

Figure S23. (a) Particle size distribution by number of nanoassemblies of Fmoc-Trp(Boc)-OH with TBTU and DIEA; (b) Particle size distribution by scattering intensity ---------------------------- 20

Figure S24. (a) Particle size distribution by number of nanoassemblies of Fmoc-Val-OH with TBTU and DIEA; (b) Particle size distribution by scattering intensity ---------------------------- 21

3-3. Particle size of nanoassemblies -- 22

Table S2. Mode diameter of Fmoc-amino acid nanoassemblies with DMT-MM and NMM. ---22

Table S3. Mode diameter of Fmoc-amino acid nanoassemblies with TBTU and DIEA. -------- 22

4. Preparation of pre-loading resin

4-1. Fmoc-Phe-Rink amide TentaGel resin -- 23

5. Characterization of peptides

5-1. HPLC anaylsys -- 23

4-2. Mass analysis -- 23

5-2. Leu-Enkephalin amide -- 24

Figure S25. Analytical HPLC profile of Leu-enkephalin amide obtained by ASPPS using nanoassemblies of Fmoc-amino acids with DMTMM and NMM. --------------------------------- 24

Figure S26. (a) Analytical HPLC profile of Leu-enkephalin amide obtained by ASPPS using nanoassemblies of Fmoc-amino acids with TBTU and DIEA; (b) ESI-MS(TOF) spectrum ---- 25

Figure S27. (a) Analytical HPLC profile of Leu-enkephalin amide obtained by ASPPS using nanoassemblies of Fmoc-amino acids with TATU and DIEA; (b) ESI-MS(TOF) spectrum ----- 26

Figure S28. (a) Analytical HPLC profile of Leu-enkephalin amide obtained by ASPPS using nanoassemblies of Fmoc-amino acids with TCTU and DIEA; (b) ESI-MS(TOF) spectrum ---- 27

5-3. Dermorphine -- 28

Figure S29. (a) Analytical HPLC profile of dermorphin obtained by ASPPS using nanoassemblies of Fmoc-amino acids with TBTU and DIEA; (b) ESI-MS(TOF) spectrum ----------------------- 28

Figure S30. (a) Analytical HPLC profile of dermorphin obtained by ASPPS using nanoassemblies of Fmoc-amino acids with TATU and DIEA; (b) ESI-MS(TOF) spectrum ------------------------29

5-4. β-Endorphin -- 30

Figure S31. (a) Analytical HPLC profile of β-endorphin (21-31) obtained by ASPPS using nanoassemblies of Fmoc-amino acids with TBTU and DIEA; (b) ESI-MS(TOF) spectrum ---- 30

Figure S32. (a) Analytical HPLC profile of β-endorphin (21-31) obtained by ASPPS using nanoassemblies of Fmoc-amino acids with TBTU and DIEA; (b) ESI-MS(TOF) spectrum; (c) Expanded view of the MS spectrum supporting the assignment of deletion-related peaks ------ 31

Figure S33. Analytical HPLC profile of purified β-endorphin obtained by ASPPS using nanoassemblies of Fmoc-amino acids with TBTU and DIEA. ------------------------------------- 32

Figure S34. (a) Analytical HPLC profile of β-endorphin obtained by conventional SPPS in DMF employing Fmoc strategy; (b) ESI-MS(TOF) spectrum -- 33

6. References --34

1. Side-by-side comparison of ASPPS approaches

Table S1. Side-by-side comparison of ASPPS approaches: nanoassembly-enabled ASPPS (this work) versus salt-based molecular solubilization (e.g., Wellings et al., 2026).

Dimension	**This work: Nanoassembly-enabled ASPPS (in-water, DMF-free)**	Salt-based molecular solubilization approach (as reported)
State of Fmoc-AA feed	Nanoassembled, non-molecularly dissolved; maintains reactive interfacial microenvironments that facilitate rapid coupling and stable operation	Molecularly dissolved via salt-assisted solubilization
Evidence of state	Tyndall effect and t number-weighted DLS, indicating persistent nanoscale assemblies under reaction conditions	Optical clarity/solubility as primary indicator; detailed aggregation state as reported
Reaction medium (coupling)	Water; additive-light (no surfactants); activator + base within nanoassemblies	Water with salt-enabled solubilization of Fmoc-AA building blocks
Conceptual basis	Heterogeneous nanoassembly-mediated reaction field (interfacial reactivity-driven)	Homogeneous molecular dissolution (solubility-driven)
Coupling reagents validated	DMT-MM; uronium reagents: TBTU, TATU, TCTU (HBTU slower under aqueous conditions)	Uronium-type reagents compatible with dissolved feeds (as reported)
Base	DIEA or NMM	As reported in original protocol
Deprotection solvent	20% piperidine/EtOAc (fully DMF-free)	Varies by implementation; DMF-free status depends on protocol
Supports	Rink-amide TentaGel (pre-loaded start); initial-loading method (SI Section 2)	As reported; not necessarily constrained to pre-loaded start
Operation mode	Commercial MW synthesizer; automation-ready feeds (stable, clog-free), enabling direct translation to existing SPPS workflows	Primarily batch/manual as reported; automation-readiness not central focus
Typical coupling time	Consistently rapid coupling (≤5 min at 75 °C, MW) without need for solubilizing salts (except HBTU)	Efficient coupling under salt-solubilized conditions (reaction times vary depending on system)

Temperature / energy input	75 °C (MW)	As reported
Longest sequence	β-Endorphin (31 aa) under aqueous coupling; crude purity 13.1% (220 nm)	As reported in original publication
Green chemistry posture	Fully DMF-free coupling; minimal additives; water-based system compatible with green chemistry principles and regulatory trends.	DMF replacement during coupling via salt solubilization; overall environmental profile depends on protocol details
Known limitations / sensitivities	HBTU slower under aqueous nanoassembly conditions; sequence-dependent difficult steps (ΔThr(8), ΔGln(11)/ΔThr(12)); minor +139 Da species (DMT-MM) mitigable by mild work-up, indicating controllable side reactions	Scope governed by salt-solubility of building blocks; performance depends on salt–substrate compatibility

* Comparator column summarizes general characteristics as reported for salt-based solubilization (e.g., Wellings et al., 2026). For exact experimental details, see the original publication.

2. Coupling reagents

2-1. Structure of coupling reagents used in this study

DMT-MM: 4-(4,6-Dimethoxy-1,3,5-triazin-2-yl)-4-methylmorpholinium chloride

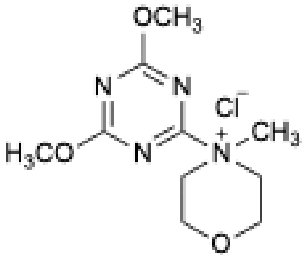

TBTU: 2-(1H-Benzotriazole-1-yl)-1,1,3,3-tetramethylaminium tetrafluoroborate

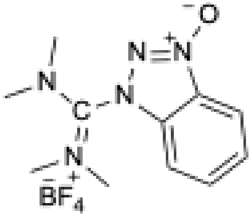

TATU: 1-[Bis(dimethylamino)methylene]-1H-1,2,3-triazolo[4,5-b]pyridinium 3-oxide tetrafluoroborate

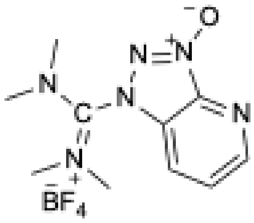

TCTU: 1-[Bis(dimethylamino)methylen]-5-chlorobenzotriazolium 3-oxide tetrafluoroborate

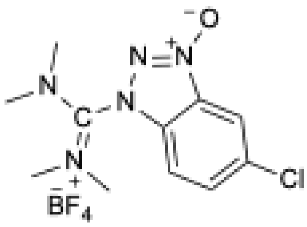

HBTU: 1-((Dimethylamino)(dimethyliminio)methyl)-1H-benzo[d][1,2,3]triazole 3-oxide hexafluorophosphate

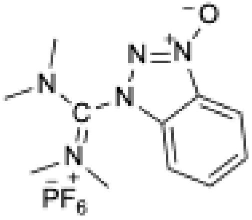

2. Dynamic light scattering (DLS) analysis[1, 2)]

2-1. DLS Measurement and Data Interpretation

This section describes how hydrodynamic diameters and polydispersity indices (PdI) were obtained using dynamic light scattering (DLS) and explains how number- and intensity-weighted distributions were interpreted.[1,2)] It also explains why, when the intensity distribution was distorted by large, reversible clusters, the mode diameter was determined from the number-weighted distribution (i.e. the smaller, more stable peak).

2-1-1. Measurement and Analysis

Method: DLS measurements were analyzed using the cumulant method to obtain the Z-average hydrodynamic diameter and polydispersity index (PdI) from the autocorrelation function.

PdI interpretation[3,4)]: A PdI value of $\leq$0.3 indicates relatively uniform populations and satisfactory cumulant fits. A PdI value greater than 0.3 signals pronounced heterogeneity and/or deviations of the autocorrelation function from the cumulant approximation.

Distributions used: The instrument software reported intensity-weighted, volume-weighted, and number-weighted size distributions. However, as scattering intensity increases rapidly with particle size (intensity $\propto d^6$), intensity-weighted distributions may overemphasize larger species, even when they constitute only a small proportion of the sample.[5)]

2-1-2. Decision Rule for Mode Diameter[1,6)]

Primary rule: The mode diameter was determined from the number-weighted distribution, as this best reflects the dominant particle population (i.e. discrete nanoassemblies) in the sample.

If the intensity shows a larger peak: If the intensity-weighted distribution exhibited a secondary peak in the larger size range, we tested dilution and brief ultrasonication. A marked decrease in the larger peak, accompanied by a shift towards the smaller peak, was taken as evidence that the larger species were transient clusters or pseudo-aggregates arising from weak interparticle interactions rather than permanent aggregates. In such cases, the smaller, stable peak observed in the number distribution that persisted after treatment was adopted as the mode diameter.

2-1-3. Representative Examples

Figure S1. Fmoc-DAla-OH nanoassemblies

(a) Number-weighted distribution revealed two populations with a dominant peak at ~79 nm (Peak 1: 358.5 nm, 1.1% by number; Peak 2: 79.89 nm, 98.9% by number). The cumulant analysis gave Z-average = 400.6 nm and PdI = 0.404, indicating heterogeneity consistent with the minor second population.

(b) Intensity-weighted distribution (before ultrasonication) showed a bimodal profile with a large peak

at ~422.8 nm (Peak 1: 422.8 nm, 66.2% by intensity) and a smaller peak at ~112.6nm (Peak 2: 112.6 nm, 33.8% by intensity). The PdI = 0.404 and the strong larger peak are consistent with intensity bias toward bigger species.

(c) Intensity-weighted distribution (after 5 min ultrasonication) shifted toward the smaller size with Peak ~102.0 nm (100% by intensity), the larger peak disappearing that the larger species were reversible clusters rather than permanent aggregates.

Interpretation and mode diameter: Because the larger-intensity peak was reversible and the smaller population was dominant in the number-weighted distribution, the mode diameter for Fmoc-DAla-OH nanoassemblies was determined from the number-weighted distribution at ~79 nm (the characteristic size of the discrete nanoassemblies).

(a)

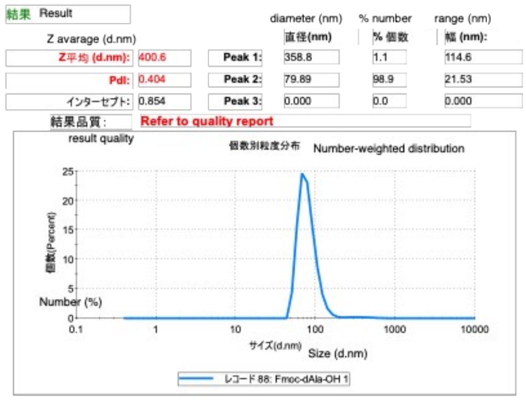

(b)

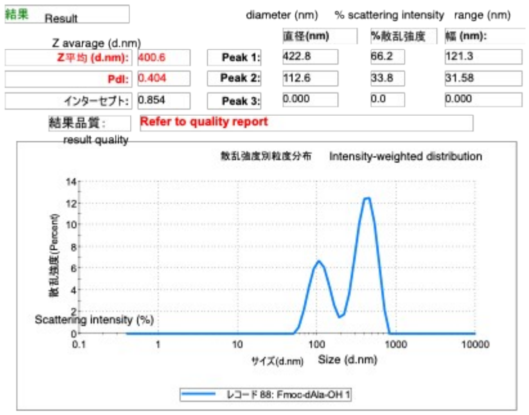

(c)

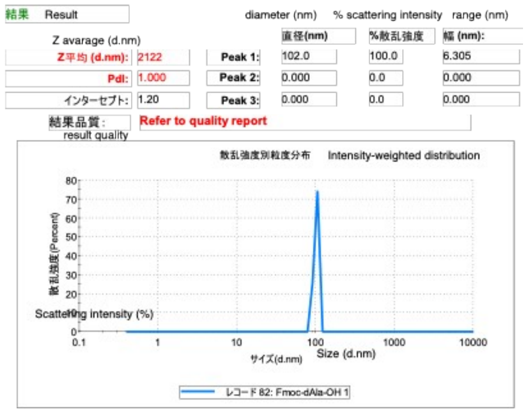

Figure S1. (a) Particle size distribution (number-weighted) for nanoassemblies of Fmoc-DAla-OH with TBTU and DIEA; (b) Particle size distribution (intensity-weighted) before ultrasonication; (c) After 5 min ultrasonication, the intensity-weighted distribution.

3-2. Particle size distribution for nanoassemblies

3-2-1. Particle size distribution for nanoassemblies of Fmoc-amino acids with DMT-MM and NMM

(a) (b)

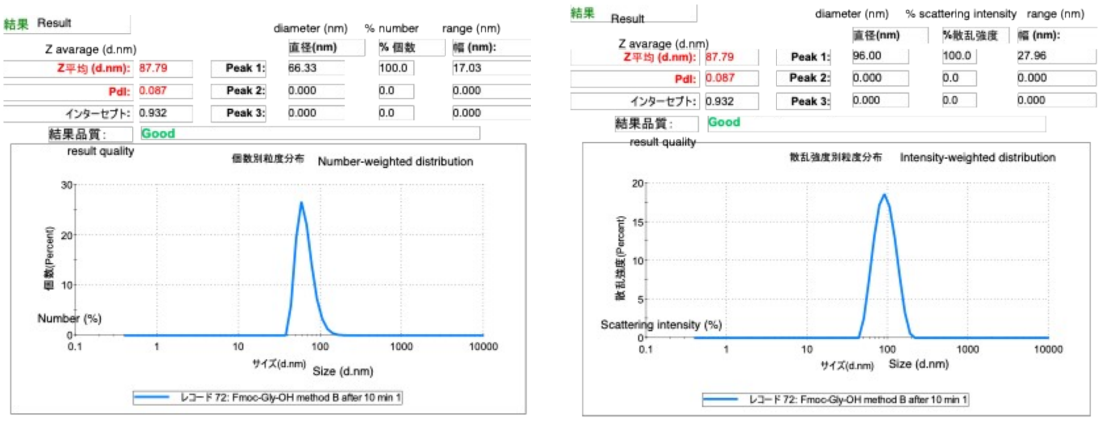

Figure S2. (a) Particle size distribution (number-weighted) for nanoassemblies of Fmoc-Gly-OH with DMT-MM and NMM showing a sharp peak at 66.3 nm (Z-average = 87.79 nm; PdI = 0.087); (b) Particle size distribution (intensity-weighted) showing a single peak at 96.0 nm due to size-dependent weighting. The mode diameter was taken from the number-weighted distribution (66.3 nm).

(a) (b)

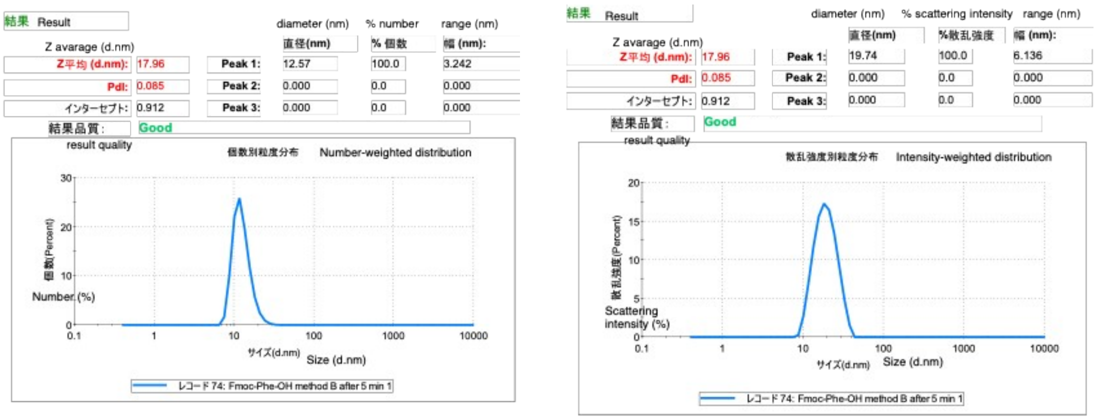

Figure S3. (a) Particle size distribution (number-weighted) for nanoassemblies of Fmoc-Phe-OH with DMT-MM and NMM showing a sharp peak at 12.57 nm (Z-average = 17.96 nm; PdI = 0.085); (b) Particle size distribution (intensity-weighted) showing a single peak at 19.74 nm due to size-dependent weighting. The mode diameter was taken from the number-weighted distribution (12.57 nm).

(a)

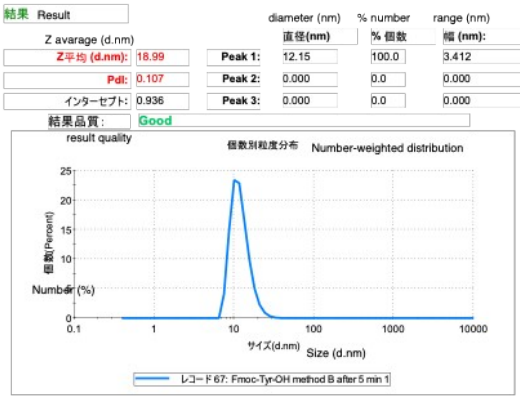

(b)

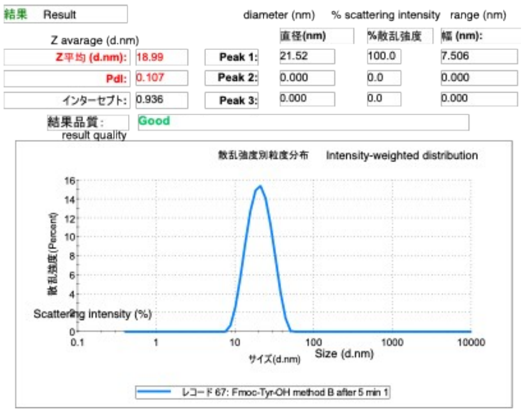

Figure S4. (a) Particle size distribution (number-weighted) for nanoassemblies of Fmoc-Tyr(tBu)-OH with DMT-MM and NMM showing a sharp peak at 12.15 nm (Z-average = 18.99 nm; PdI = 0.107); (b) Particle size distribution (intensity-weighted) showing a single peak at 21.52 nm due to size-dependent weighting. The mode diameter was taken from the number-weighted distribution (12.15 nm).

3-2-2. Particle size distribution for nanoassemblies of Fmoc-amino acids with TBTU and DIEA

(a)

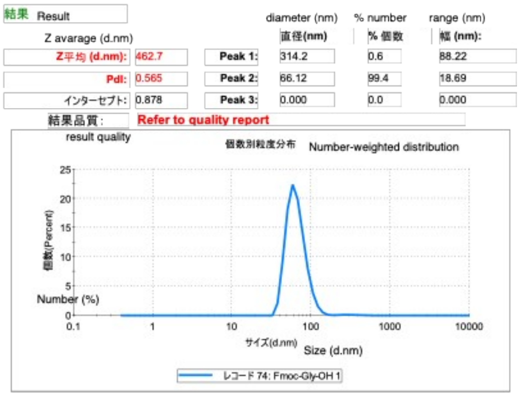

(b)

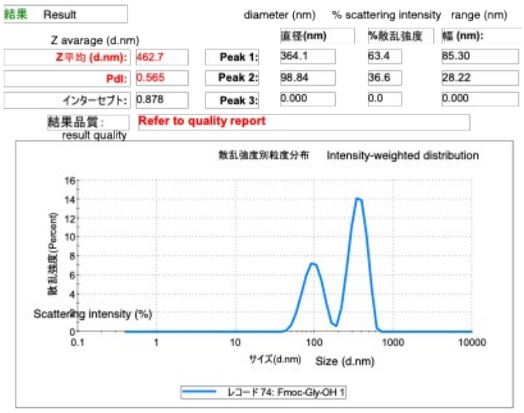

Figure S5. (a) Particle size distribution (number-weighted) for nanoassemblies of Fmoc-Gly-OH with TBTU and DIEA showing a dominant peak at 66.12 nm (Z-average = 462.7 nm; PdI = 0.565); (b) Particle size distribution (intensity-weighted) before ultrasonication exhibiting a bimodal profile with peaks at 364.1nm and 98.84 nm, consistent with intensity bias toward larger species. The mode diameter was determined from the number-weighted distribution (66.12 nm).

(a) (b)

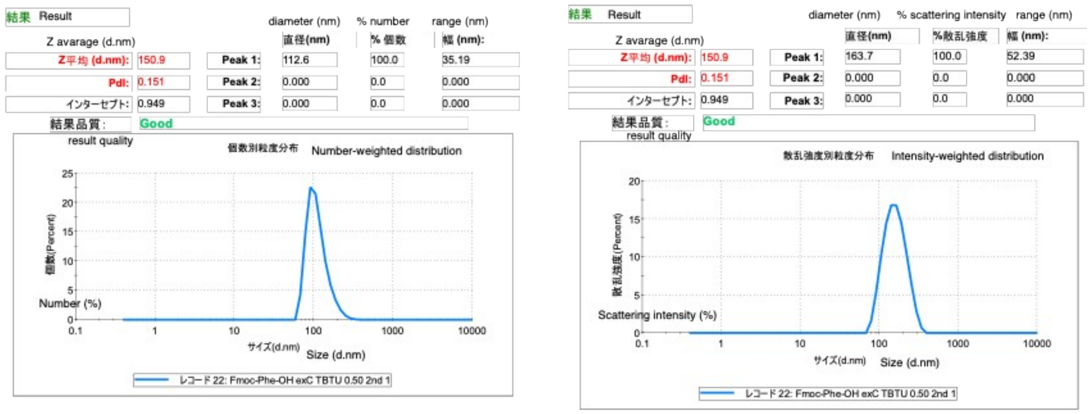

Figure S6. (a) Particle size distribution (number-weighted) for nanoassemblies of Fmoc-Phe-OH with TBTU and DIEA showing a sharp peak at 112.6 nm (Z-average = 150.9 nm; PdI = 0.151); (b) Particle size distribution (intensity-weighted) showing a single peak at 163.7 nm due to size-dependent weighting. The mode diameter was taken from the number-weighted distribution (63.21 nm).

(a) (b)

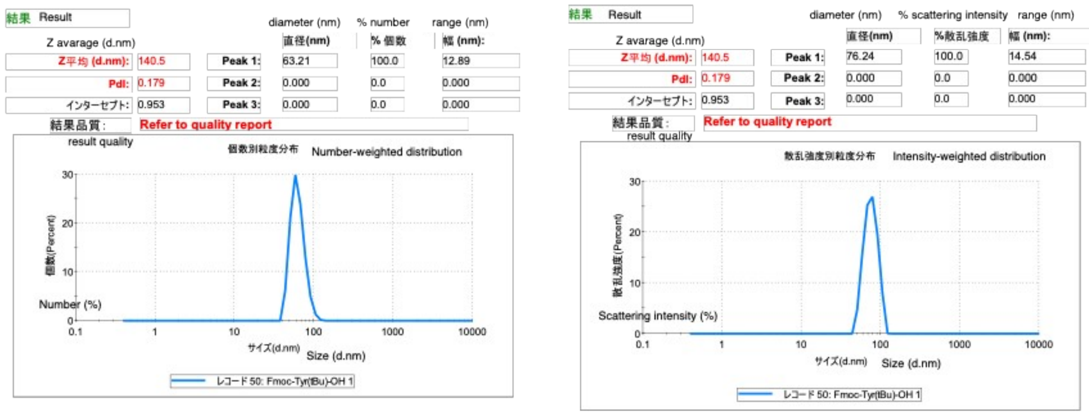

Figure S7. (a) Particle size distribution (number-weighted) for nanoassemblies of Fmoc-Tyr(tBu)-OH with TBTU and DIEA showing a sharp peak at 63.21 nm (Z-average = 140.5 nm; PdI = 0.179); (b) Particle size distribution (intensity-weighted) showing a single peak at 76.24 nm due to size-dependent weighting. The mode diameter was taken from the number-weighted distribution (112.6 nm).

(a) (b)

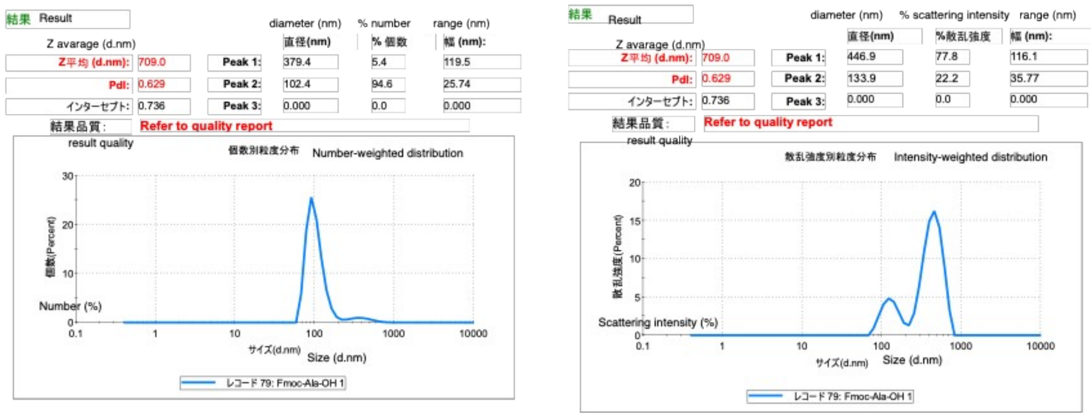

Figure S8 (a) Particle size distribution (number-weighted) for nanoassemblies of Fmoc-Ala-OH with TBTU and DIEA showing a dominant peak at 102.4 nm (Z-average = 709.0 nm; PdI = 0.629); (b) Particle size distribution (intensity-weighted) before ultrasonication exhibiting a bimodal profile with peaks at 446.9 nm and 133.9 nm, consistent with intensity bias toward larger species. The mode diameter was determined from the number-weighted distribution (102.4 nm).

(a) (b)

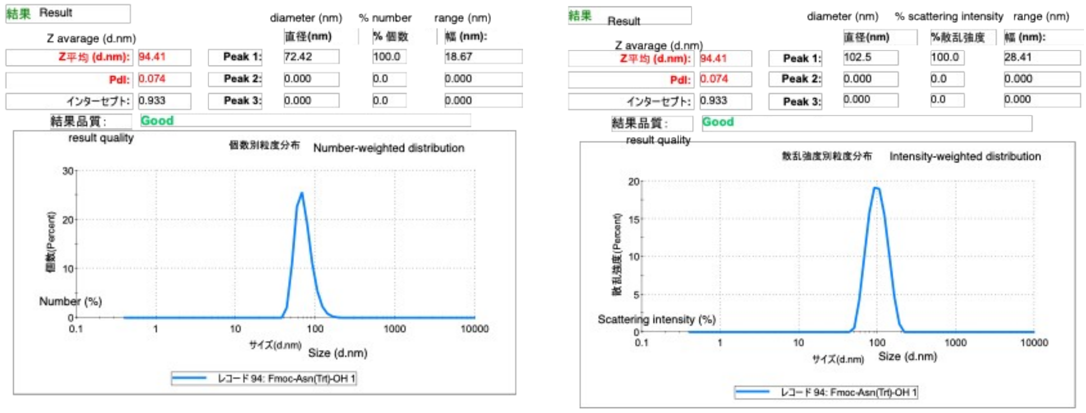

Figure S9. (a) Particle size distribution (number-weighted) for nanoassemblies of Fmoc-Asn(Trt)-OH with TBTU and DIEA showing a sharp peak at 72.42 nm (Z-average = 94.41 nm; PdI = 0.074); (b) Particle size distribution (intensity-weighted) showing a single peak at 102.5 nm due to size-dependent weighting. The mode diameter was taken from the number-weighted distribution (72.42 nm).

(a) (b)

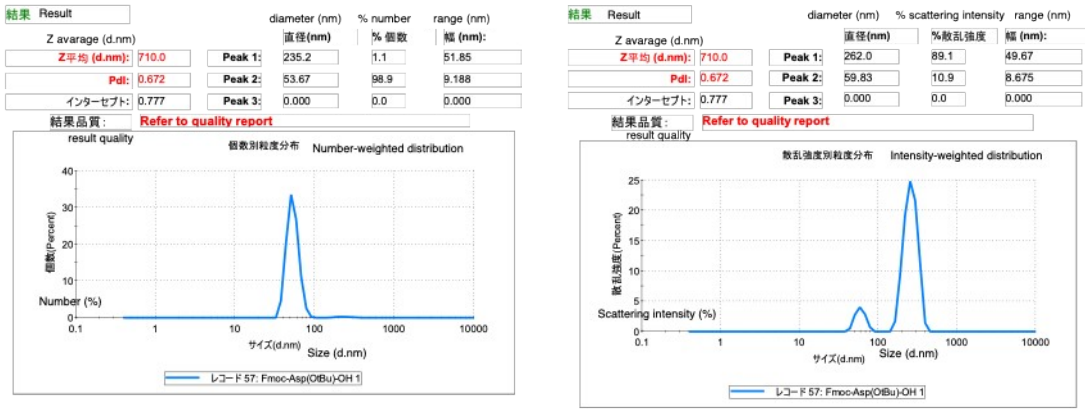

Figure S10. (a) Particle size distribution (number-weighted) for nanoassemblies of Fmoc-Asp(OtBu)-OH with TBTU and DIEA showing a dominant peak at 53.67nm (Z-average = 710.0 nm; PdI = 0.672); (b) Particle size distribution (intensity-weighted) before ultrasonication exhibiting a bimodal profile with peaks at 262.0 nm and 59.83 nm, consistent with intensity bias toward larger species. The mode diameter was determined from the number-weighted distribution (53.67 nm).

(a) (b)

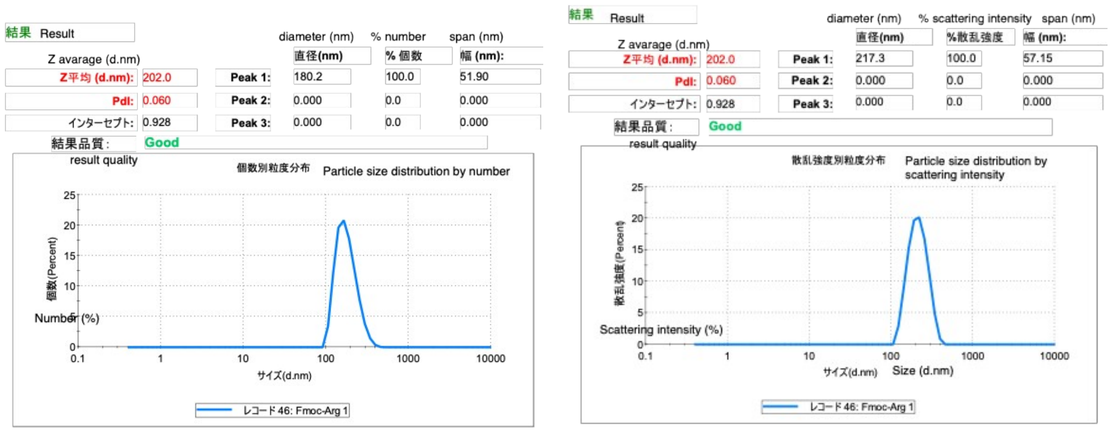

Figure S11 (a) Particle size distribution (number-weighted) for nanoassemblies of Fmoc-Arg(Pbf)-OH with TBTU and DIEA showing a sharp peak at 180.2nm (Z-average = 202.0 nm; PdI = 0.060); (b) Particle size distribution (intensity-weighted) showing a single peak at 217.3 nm due to size-dependent weighting. The mode diameter was taken from the number-weighted distribution (180.2 nm).

(a) (b)

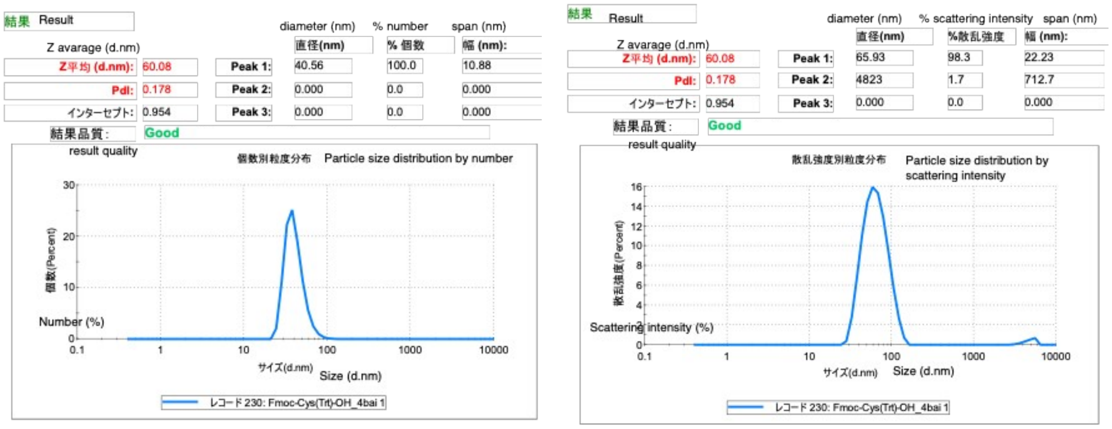

Figure S12. (a) Particle size distribution (number-weighted) for nanoassemblies of Fmoc-Cys(Trt)-OH with TBTU and DIEA showing a sharp peak at 40.56 nm (Z-average = 60.06 nm; PdI = 0.178); (b) Particle size distribution (intensity-weighted) showing a single peak at 65.93 nm due to size-dependent weighting. The mode diameter was taken from the number-weighted distribution (40.56 nm).

(a) (b)

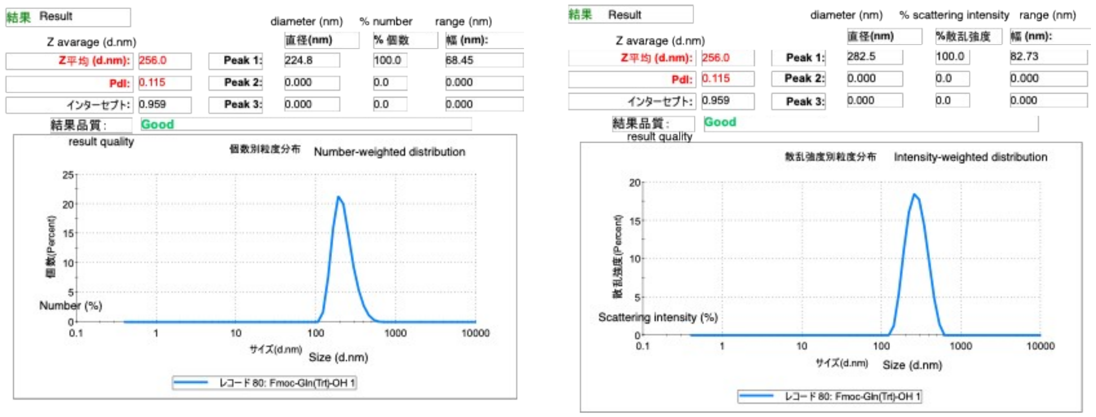

Figure S13. (a) Particle size distribution (number-weighted) for nanoassemblies of Fmoc-Gln(Trt)-OH with TBTU and DIEA showing a sharp peak at 224.8 nm (Z-average = 256.0 nm; PdI = 0.115); (b) Particle size distribution (intensity-weighted) showing a single peak at 282.5 nm due to size-dependent weighting. The mode diameter was taken from the number-weighted distribution (224.8 nm).

(a)

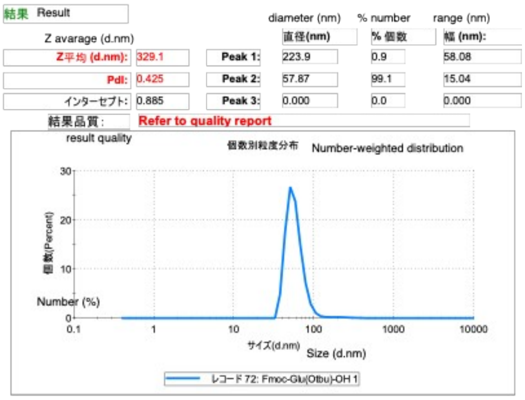

(b)

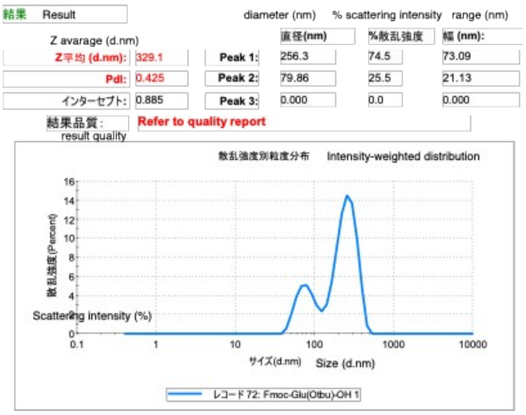

Figure S14. (a) Particle size distribution (number-weighted) for nanoassemblies of Fmoc-Glu(OtBu)-OH with TBTU and DIEA showing a dominant peak at 57.87nm (Z-average =329.1 nm; PdI = 0.425); (b) Particle size distribution (intensity-weighted) before ultrasonication exhibiting a bimodal profile with peaks at 256.3 nm and 79.86 nm, consistent with intensity bias toward larger species.

(a)

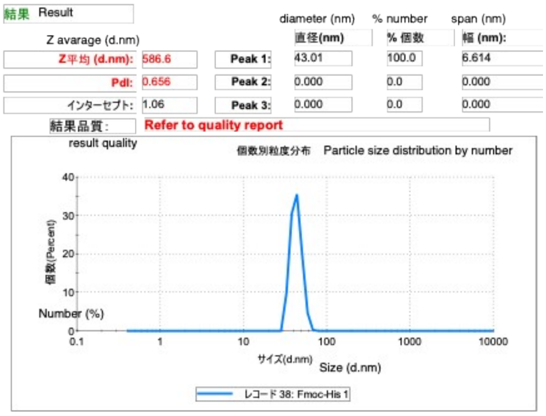

(b)

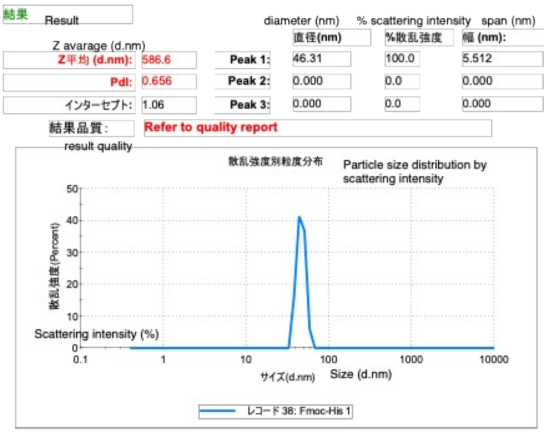

Figure S15. (a) Particle size distribution (number-weighted) for nanoassemblies of Fmoc-His(Boc)-OH with TBTU and DIEA showing a sharp peak at 43.01nm (Z-average =586.6 nm; PdI = 0.656); (b) Particle size distribution (intensity-weighted) showing a single peak at 46.31 nm due to size-dependent weighting. The mode diameter was taken from the number-weighted distribution (43.01 nm).

(a)

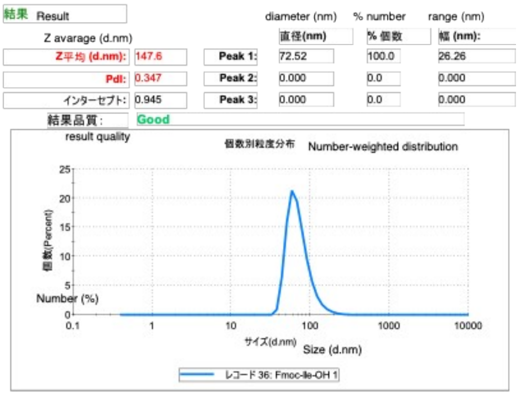

(b)

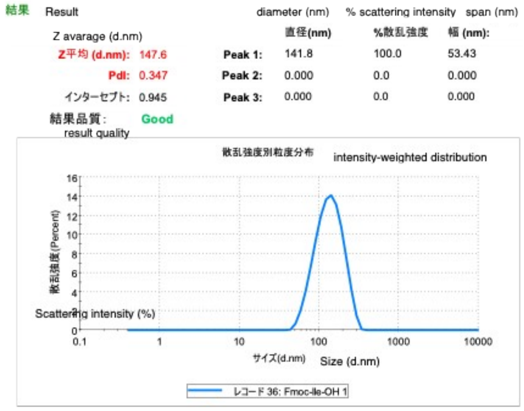

Figure S16. (a) Particle size distribution (number-weighted) for nanoassemblies of Fmoc-Ile-OH with TBTU and DIEA showing a sharp peak at 72.52 nm (Z-average = 147.6 nm; PdI = 0.347); (b) Particle size distribution (intensity-weighted) showing a single peak at 141.8 nm due to size-dependent weighting. The mode diameter was taken from the number-weighted distribution (72.52 nm).

(a)

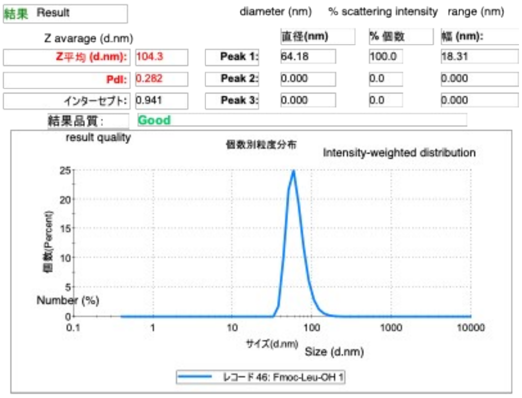

(b)

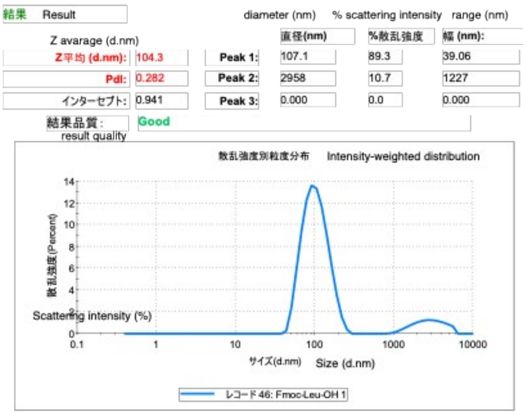

Figure S17. (a) Particle size distribution (number-weighted) for nanoassemblies of Fmoc-Leu-OH with TBTU and DIEA showing a dominant peak at 64.18nm (Z-average = 104.3 nm; PdI = 0.282); (b) Particle size distribution (intensity-weighted) before ultrasonication exhibiting a bimodal profile with peaks at 107.1nm and 2958 nm, consistent with intensity bias toward larger species. The mode diameter was determined from the number-weighted distribution (64.18 nm).

(a) (b)

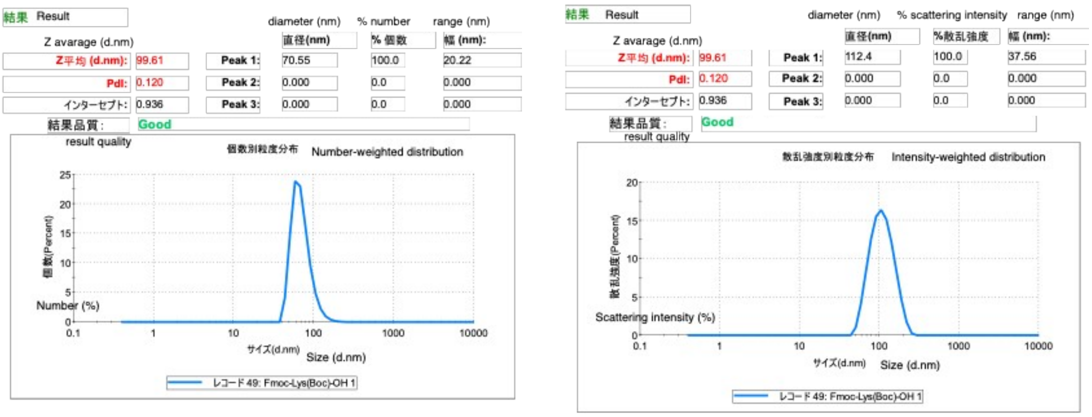

Figure S18. (a) Particle size distribution (number-weighted) for nanoassemblies of Fmoc-Lys(Boc)-OH with TBTU and DIEA showing a sharp peak at 70.55 nm (Z-average = 99.61 nm; PdI = 0.120); (b) Particle size distribution (intensity-weighted) showing a single peak at 112.4 nm due to size-dependent weighting. The mode diameter was taken from the number-weighted distribution (70.55 nm).

(a) (b)

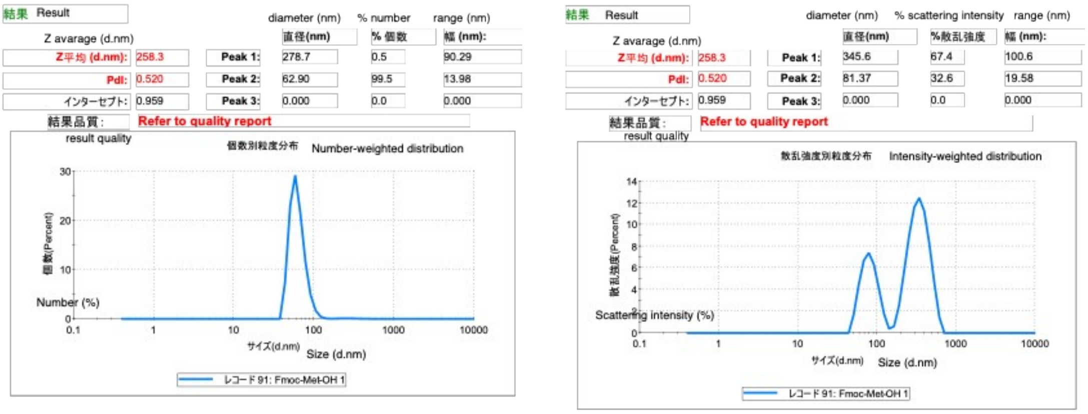

Figure S19. (a) Particle size distribution (number-weighted) for nanoassemblies of Fmoc-Met-OH with TBTU and DIEA showing a dominant peak at 62.90 nm (Z-average = 258.3 nm; PdI = 0.520); (b) Particle size distribution (intensity-weighted) before ultrasonication exhibiting a bimodal profile with peaks at 345.6 nm and 81.37 nm, consistent with intensity bias toward larger species. The mode diameter was determined from the number-weighted distribution (62.90 nm).

(a) (b)

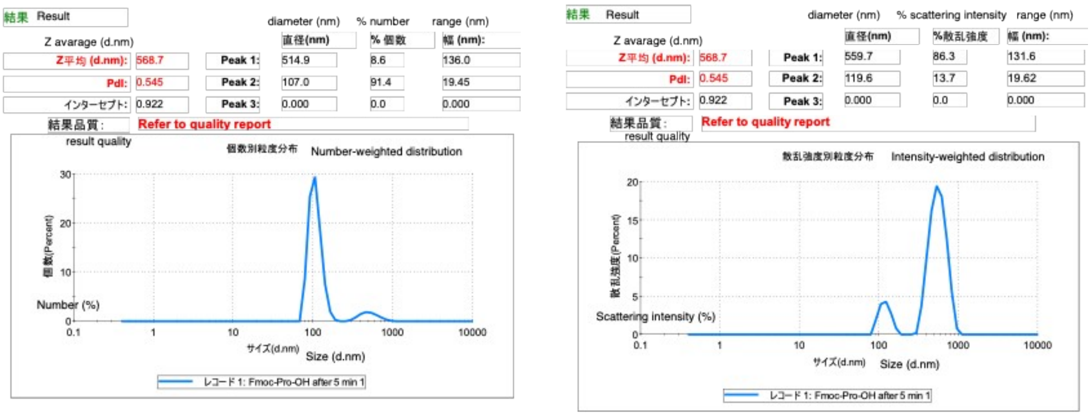

Figure S20. (a) Particle size distribution (number-weighted) for nanoassemblies of Fmoc-Pro-OH with TBTU and DIEA showing a dominant peak at 107.0 nm (Z-average = 568.7 nm; PdI = 0.545). (b) Particle size distribution (intensity-weighted) before ultrasonication exhibiting a bimodal profile with peaks at 559.7 nm and 119.6 nm, consistent with intensity bias toward larger species. The mode diameter was determined from the number-weighted distribution (107.0 nm).

(a) (b)

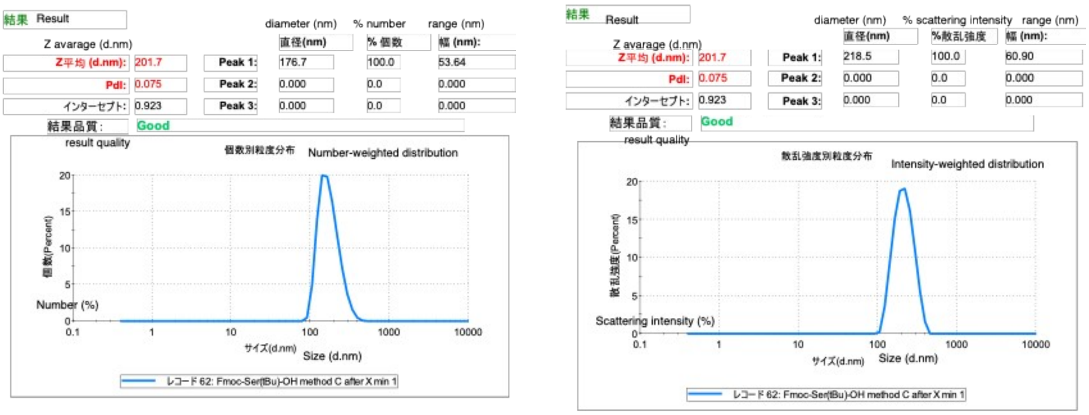

Figure S21. (a) Particle size distribution (number-weighted) for nanoassemblies of Fmoc-Ser(tBu)-OH with TBTU and DIEA showing a sharp peak at 176.7 nm (Z-average = 201.7 nm; PdI = 0.075); (b) Particle size distribution (intensity-weighted) showing a single peak at 218.5 nm due to size-dependent weighting. The mode diameter was taken from the number-weighted distribution (176.7 nm).

(a) (b)

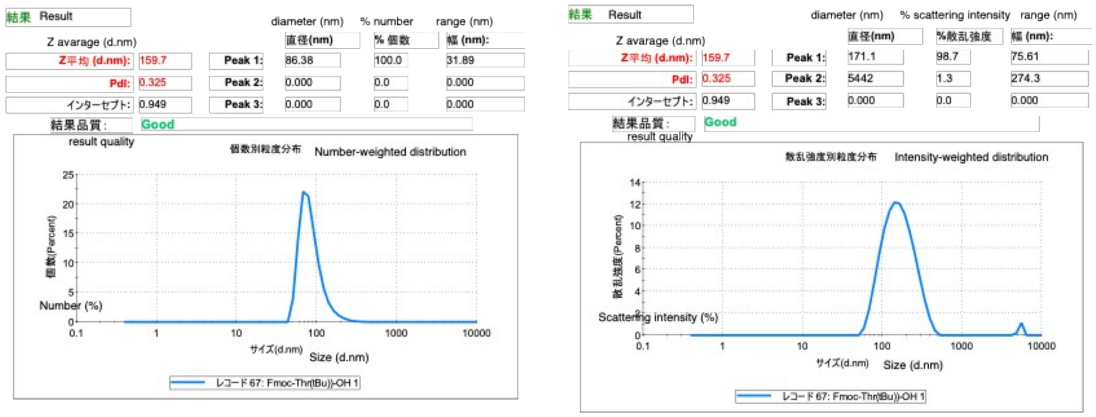

Figure S22. (a) Particle size distribution (number-weighted) for nanoassemblies of Fmoc-Thr(tBu)-OH with TBTU and DIEA showing a sharp peak at 86.38 nm (Z-average = 159.7 nm; PdI = 0.325). (b) Particle size distribution (intensity-weighted) showing a dominant peak at 171.1 nm due to size-dependent weighting. The mode diameter was taken from the number-weighted distribution (86.38 nm).

(a) (b)

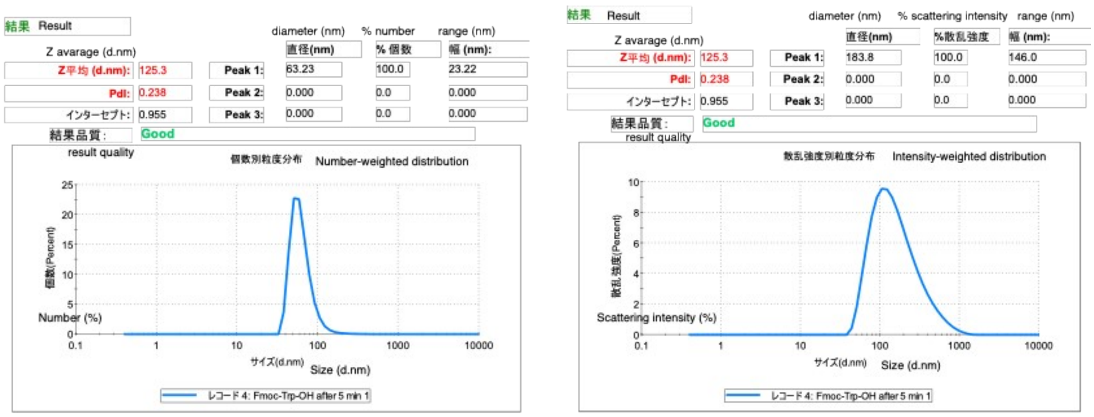

Figure S23. (a) Particle size distribution (number-weighted) for nanoassemblies of Fmoc-Trp(Boc)-OH with TBTU and DIEA showing a sharp peak at 63.23 nm (Z-average = 125.3 nm; PdI = 0.238); (b) Particle size distribution (intensity-weighted) showing a dominant peak at 183.8 nm due to size-dependent weighting. The mode diameter was taken from the number-weighted distribution (63.23 nm).

(a) (b)

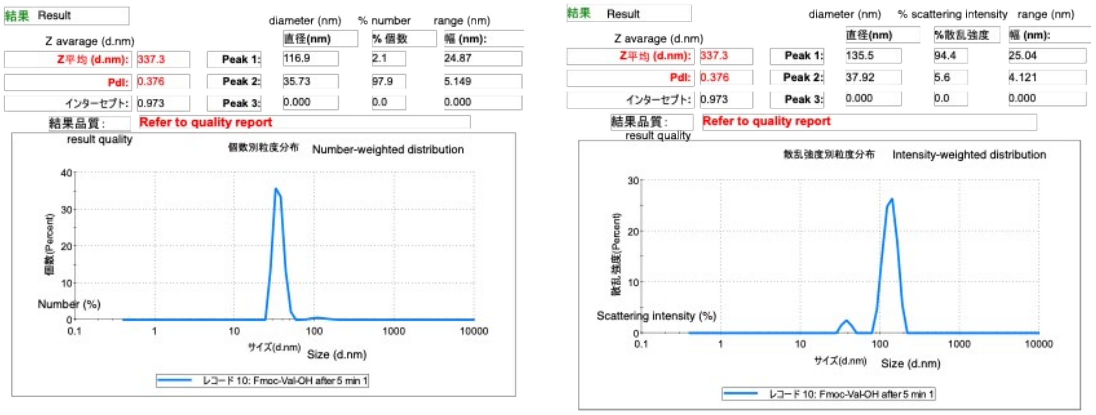

Figure S24. (a) Particle size distribution (number-weighted) for nanoassemblies of Fmoc-Val-OH with TBTU and DIEA showing a dominant peak at 35.73 nm (Z-average = 337.3 nm; PdI = 0.376); (b) Particle size distribution (intensity-weighted) before ultrasonication exhibiting a bimodal profile with peaks at 135.5 nm and 37.92 nm, consistent with intensity bias toward larger species. The mode diameter was determined from the number-weighted distribution (35.73 nm).

3-3. Particle size of nanoassemblies

Table S2. Mode diameter of Fmoc-amino acid nanoassemblies with DMT-MM and NMM.

Fmoc-amino acid	Diameter (nm)	Range (nm)	PdI
Fmoc-Gly-OH	66.3	17.03	0.087
Fmoc-Phe-OH	12.57	3.242	0.085
Fmoc-Tyr(tBu)-OH	12.15	3.412	0.107

Table S3. Mode diameter of Fmoc-amino acid nanoassemblies with TBTU and DIEA.

Fmoc-amino acid	Diameter (nm)	Range (nm)	PdI
Fmoc-Gly-OH	66.12	18.69	0.565
Fmoc-Phe-OH	112.6	35.19	0.151
Fmoc-Tyr(tBu)-OH	63.21	12.89	0.179
Fmoc-Ala-OH	102.4	25.74	0.629
Fmoc-Asn(Trt)-OH*	72.42	18.67	0.074
Fmoc-Asp(OtBu)-OH	53.67	9.188	0.672
Fmoc-DAla-OH	79.89	21.53	0.404
Fmoc-Arg(Pbf)-OH*			
Fmoc-Cys(Trt)-OH*			
Fmoc-Gln(Trt)-OH*	224.8	68.45	0.115
Fmoc-Glu(OtBu)-OH	57.87	15.04	0.425
Fmoc-His(Boc)-OH			
Fmoc-Ile-OH	72.52	26.26	0.347
Fmoc-Leu-OH	64.18	18.31	0.282
Fmoc-Lys(Boc)-OH	70.55	20.22	0.120
Fmoc-Met-OH	62.90	13.98	0.520
Fmoc-Pro-OH	107.0	19.45	0.545
Fmoc-Ser(tBu)-OH	176.7	53.64	0.075
Fmoc-Thr(tBu)-OH	86.38	31.89	0.325
Fmoc-Trp(Boc)-OH	63.23	23.22	0.238
Fmoc-Val-OH	35.73	5.149	0.376

* aqueous 10% *i*-PrOH solution was used for preparation.

4. Preparation of pre-loading resin

4-1. Preparation of Fmoc-Phe-Rink amide TentaGel resin (example)

Fmoc-Rink amide-TentaGel resin (100 mg, loading: 0.25 mmol g^{-1}, 25 μmol) was swollen in i-PrOH (5 mL) for 1 h at room temperature and then washed with EtOAc. The Fmoc group was removed using 20% piperidine in EtOAc (5 mL) for 20 min at room temperature, followed by washing with i-PrOH (5 mL × 3).

The resulting H-Rink amide-TentaGel resin was swollen in water, and aqueous nanoassemblies (6 mL, 16.7 mM with respect to Fmoc-amino acid), prepared from Fmoc-amino acid (100 μmol), TBTU (100 μmol), and DIEA (200 μmol), were coupled sequentially onto the resin. Microwave-assisted coupling reactions were performed at 75 °C for 20 min using an Initiator+ SP Wave system.

Finally, the Fmoc group was removed using 20% piperidine in EtOAc (5 mL) for 20 min at room temperature, followed by washing with i-PrOH (5 mL × 3), to afford H-Phe-Rink amide-TentaGel resin.

5. Characterization for synthetic peptides

5-1. HPLC analysis

HPLC analysis was performed using a Waters Alliance e2695 system equipped with a COSMOSIL 5C18-AR-II column (4.6 × 150 mm). Elution was carried out using a gradient of acetonitrile/water containing 0.1% trifluoroacetic acid (TFA) at a flow rate of 1.0 mL/min, with UV detection at 220 nm.

Chromatograms were processed using a uniform integration protocol. Baseline-separated peaks with a signal-to-noise ratio (S/N) ≥ 10 were integrated. Solvent front and system-related artifacts were excluded. Valley-to-valley integration was applied when the valley depth was ≥ 10% of the local peak height.

All reported crude purities are based on peak area percentages calculated under these conditions. Purity values reported in the main text are rounded to the nearest integer, while more precise values are provided in the SI.

5-2. Mass analysis

Mass spectra were recorded on an electrospray ionization quadrupole time-of-flight mass spectrometer (ESI-Q-TOF-MS, micrOTOF-Q, Bruker Daltonik GmbH). Samples were dissolved in acetonitrile/water containing 0.1% formic acid and introduced by direct infusion.

Spectra were acquired in positive ion mode over an appropriate m/z range. The observed m/z values correspond to protonated molecular ions ($[M+H]^+$) unless otherwise noted.

5-3. Leu-enkephalin amide

(a)

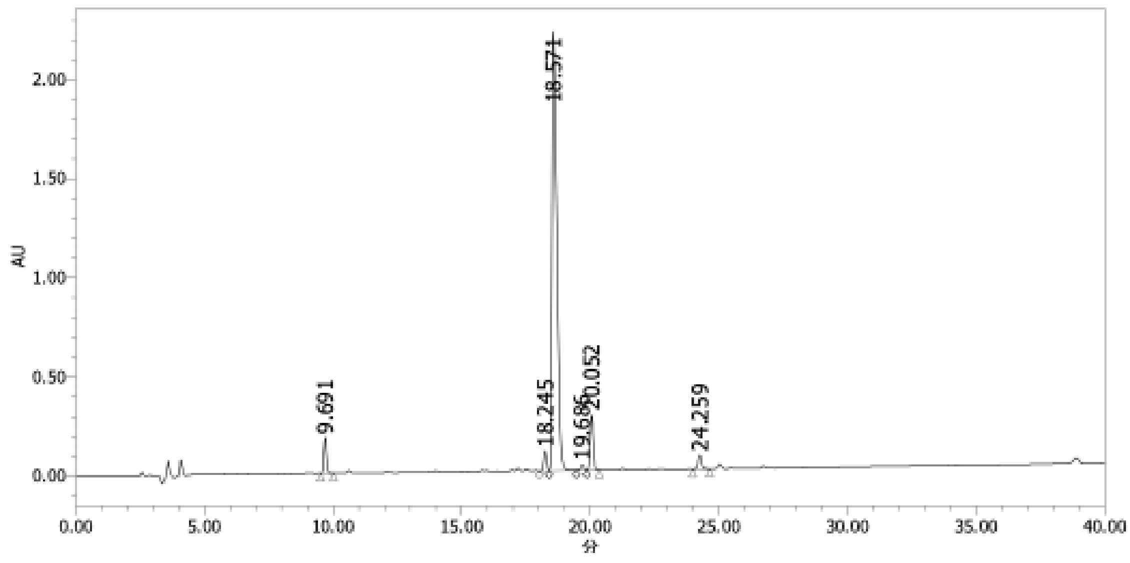

(b)

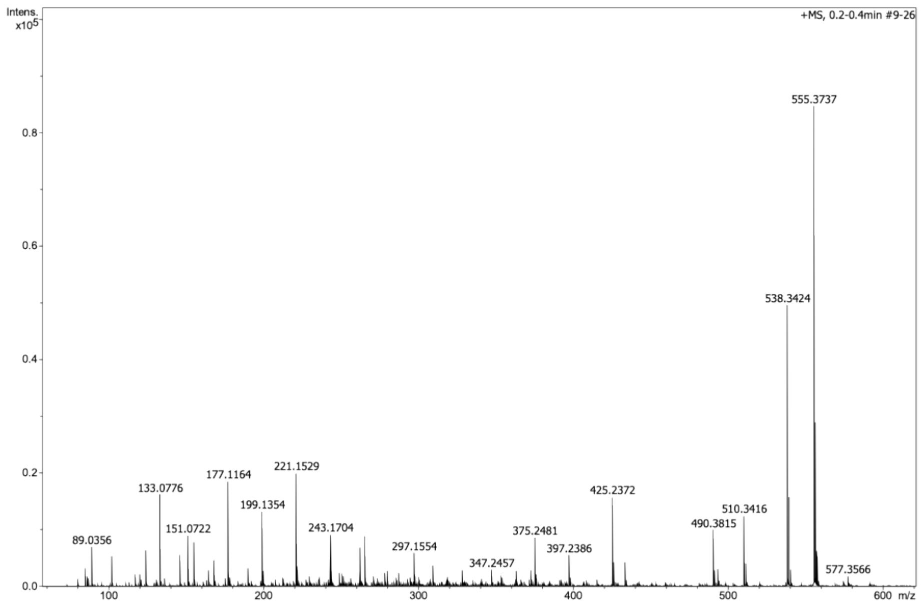

Figure S25. (a) Analytical HPLC profiles of crude Leu-enkephalin amide obtained by ASPPS employing nanoassemblies of Fmoc-amino acids with DMT-MM and NMM. Elution was carried out over 40 min at a flow rate of 1 mL/min with a linear gradient from 90:10 to 50:50 mixture of 0.1% aqueous TFA and 0.1% TFA in acetonitrile. A dominant peak was observed at a retention time of 18.6 min with a calculated purity of 84.8%; (b) ESI-MS (TOF) spectra. The observed mass was m/z 555.3737 ([M+H]$^+$), which corresponds to $C_{28}H_{39}N_6O_6$ (calculated: 555.2931).

(a)

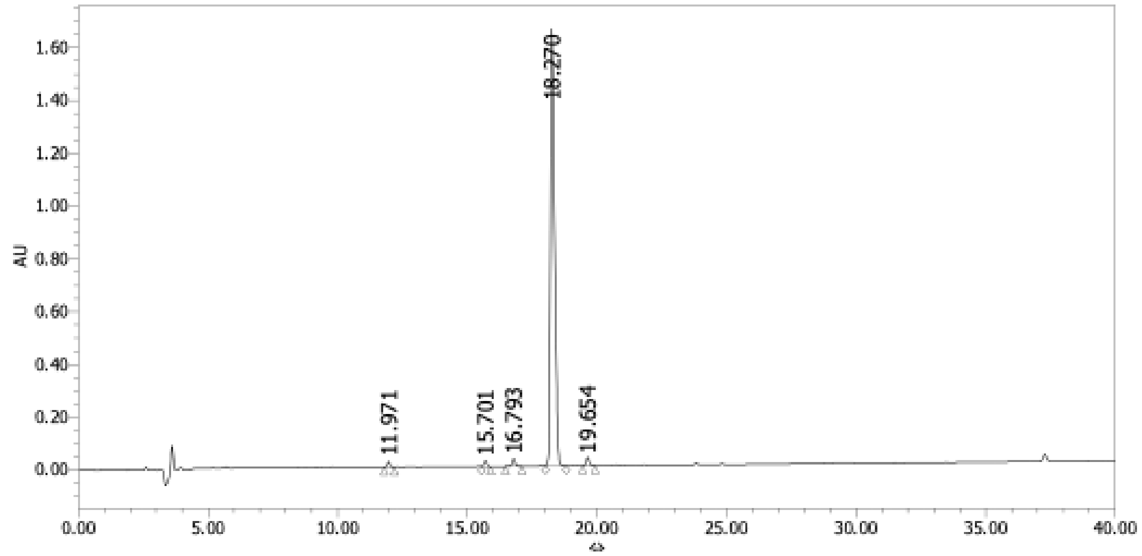

(b)

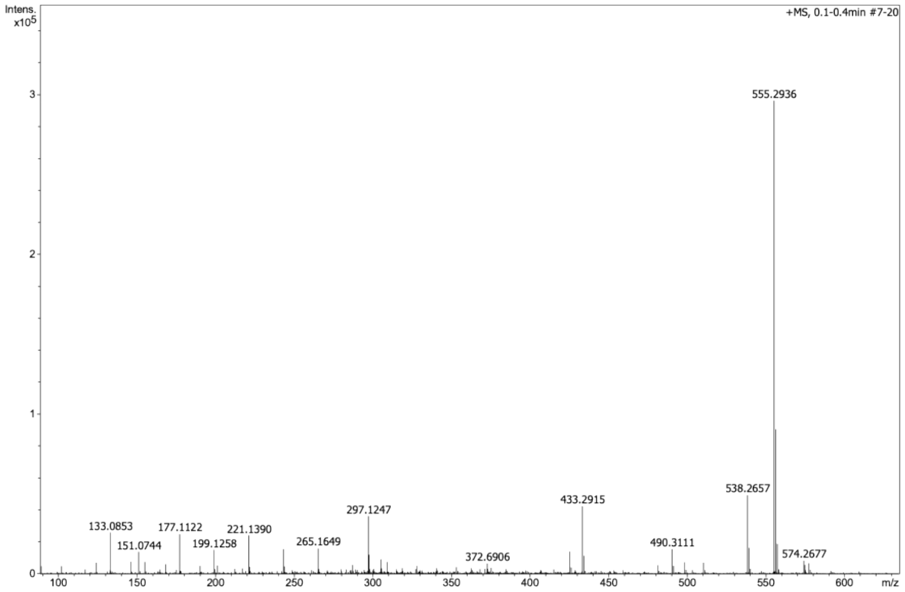

Figure S26. (a) Analytical HPLC profiles of crude Leu-enkephalin amide obtained by ASPPS employing nanoassemblies of Fmoc-amino acids with TBTU and DIEA. Elution was carried out over 40 min at a flow rate of 1 mL/min with a linear gradient from 90:10 to 50:50 mixture of 0.1% aqueous TFA and 0.1% TFA in acetonitrile. The single peak was observed at a retention time of 18.3 min with a calculated purity of 95.6%; (b) ESI-MS (TOF) spectra. The observed mass was m/z 555.2936 ($[M+H]^+$), which corresponds to $C_{28}H_{39}N_6O_6$ (calculated: 555.2931).

(a)

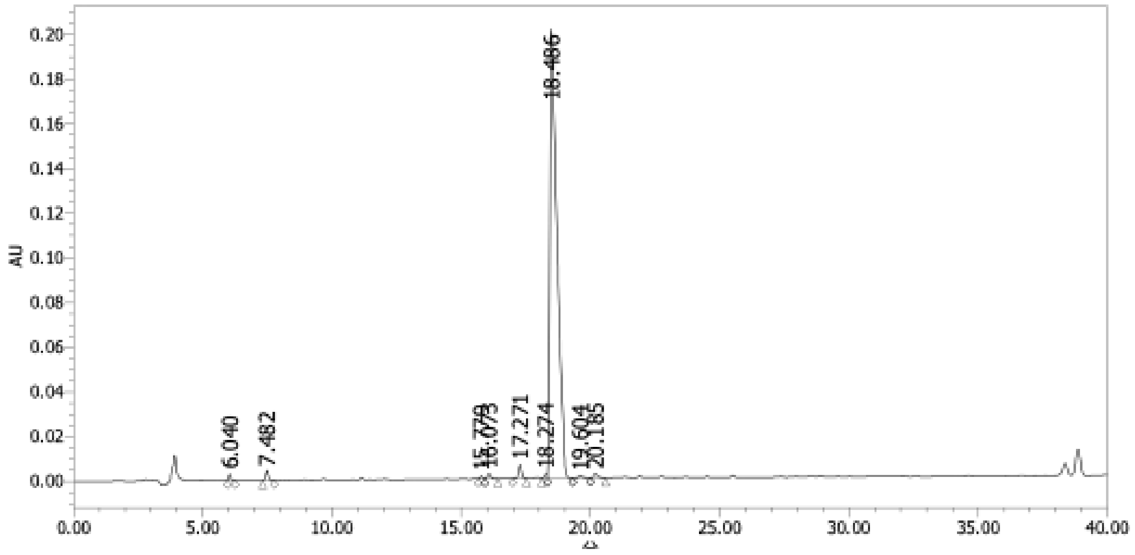

(b)

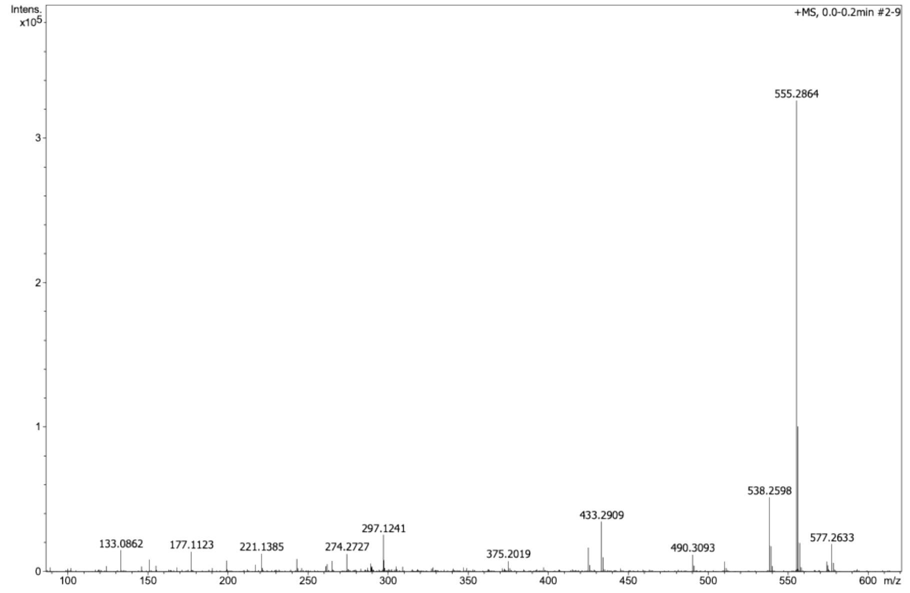

Figure S27. (A) Analytical HPLC profiles of crude Leu-enkephalin amide obtained by ASPPS employing nanoassemblies of Fmoc-amino acids with TATU and DIEA. Elution was carried out over 40 min at a flow rate of 1 mL/min with a linear gradient from 90:10 to 50:50 mixture of 0.1% aqueous TFA and 0.1% TFA in acetonitrile. A dominant peak was observed at a retention time of 18.5 min with a calculated purity of 95.4%. Later peaks at 38.3 and 38.8 min were attributed to column contamination; (b) ESI-MS (TOF) spectra. The observed mass was m/z 555.2864 ([M+H]$^+$), which corresponds to $C_{28}H_{39}N_6O_6$ (calculated: 555.2931).

(a)

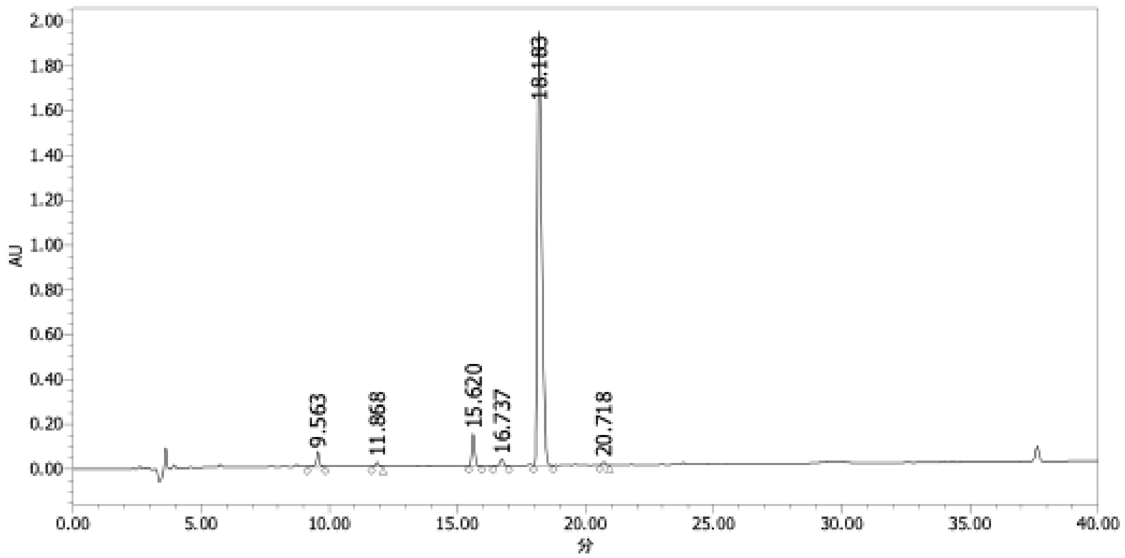

(b)

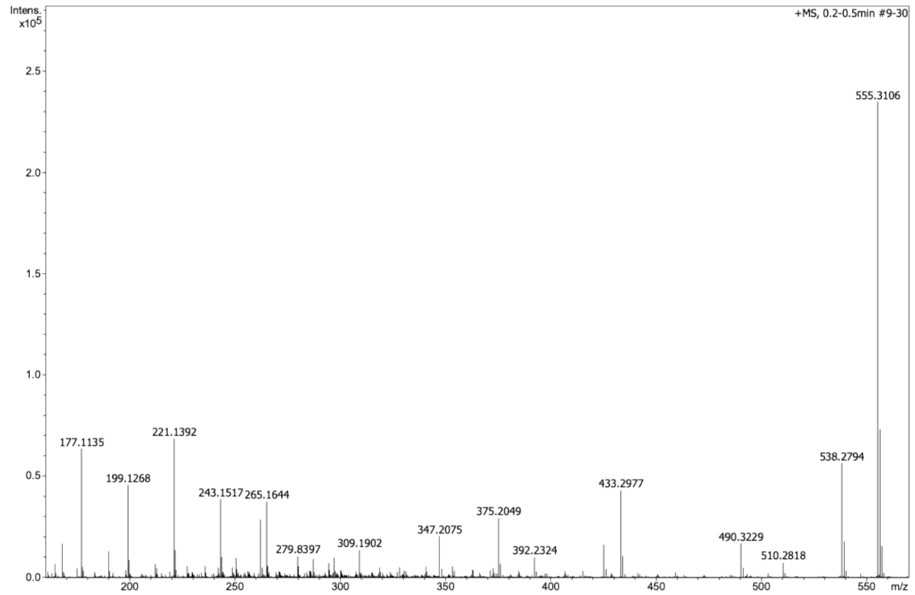

Figure S28. (a) Analytical HPLC profiles of crude Leu-enkephalin amide obtained by ASPPS employing nanoassemblies of Fmoc-amino acids with TCTU and DIEA. Elution was carried out over 40 min at a flow rate of 1 mL/min with a linear gradient from 90:10 to 50:50 mixture of 0.1% aqueous TFA and 0.1% TFA in acetonitrile. A dominant peak was observed at a retention time of 18.5 min with a calculated purity of 91.1%. Later peak at 38.5 min was attributed to column contamination; (b) ESI-MS (TOF) spectra. The observed mass was m/z 555.3106 ($[M+H]^+$), which corresponds to $C_{28}H_{39}N_6O_6$ (calculated: 555.2931).

5-4. Dermorphin

(a)

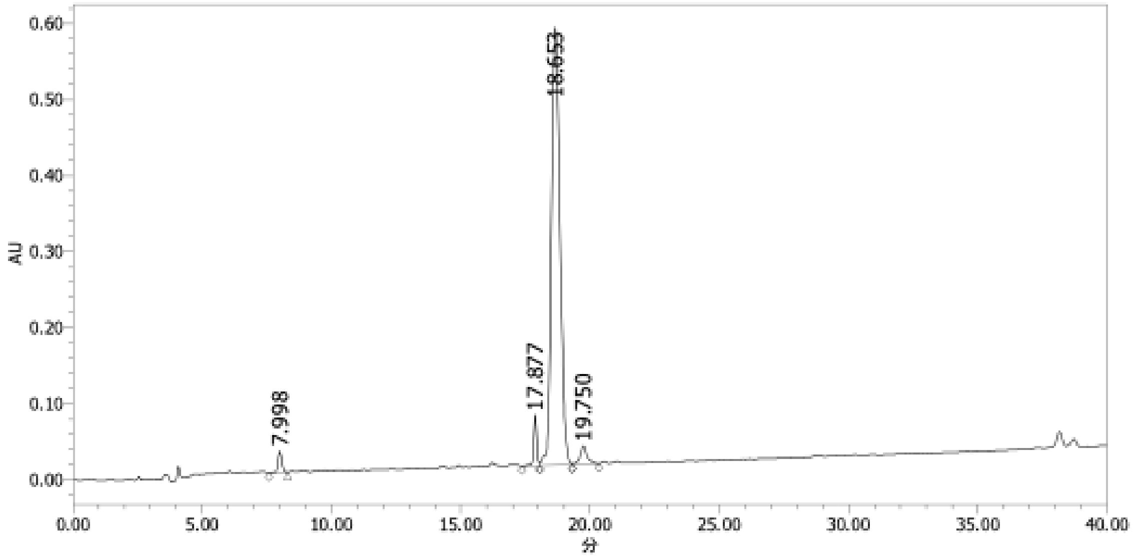

(b)

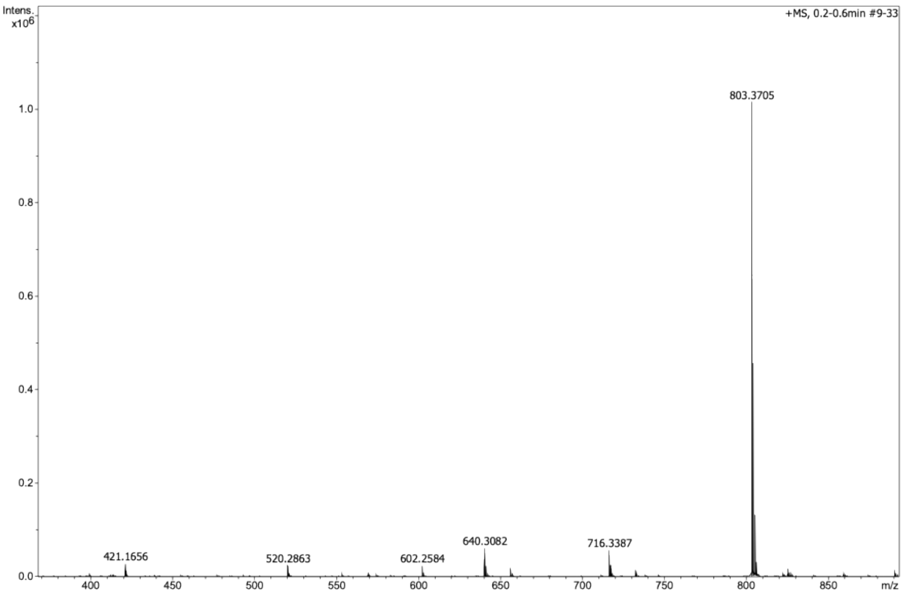

Figure S29. (a) Analytical HPLC profiles of crude dermorphin obtained by ASPPS employing nanoassemblies of Fmoc-amino acids with TBTU and DIEA. Elution was carried out over 40 min at a flow rate of 1 mL/min with a linear gradient from 90:10 to 50:50 mixture of 0.1% aqueous TFA and 0.1% TFA in acetonitrile. Two peaks were observed at retention time of 17.9 min (*cis* isomer) and18.6 min (*trans* isomer) with a calculated purity of 94.7%; (b) ESI-MS (TOF) spectra. The observed mass was m/z 803.3721 ([M+H]$^+$), which corresponds to $C_{40}H_{51}N_8O_{10}$ (calculated: 803.3728).

(a)

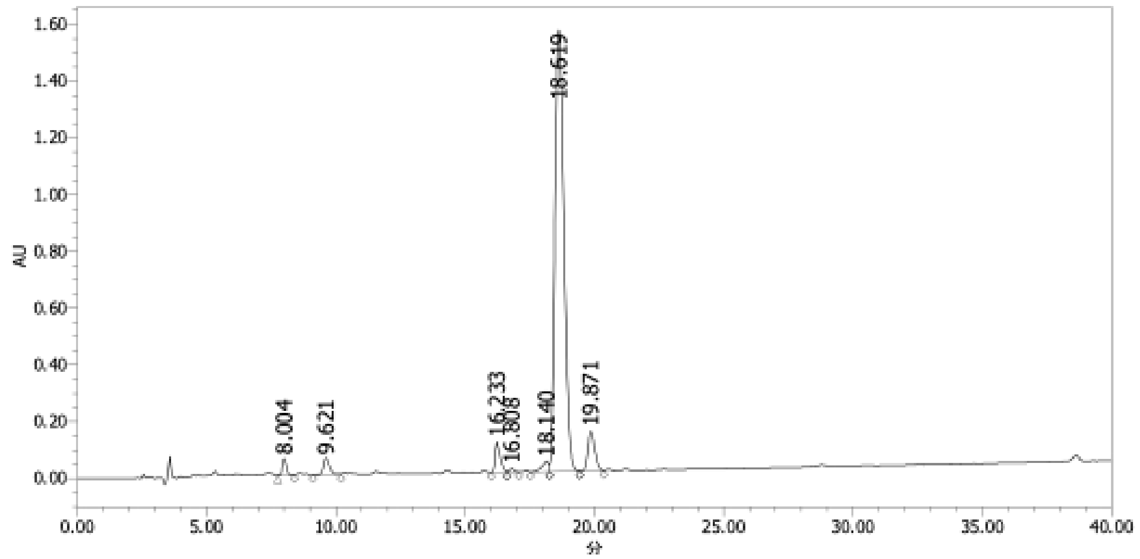

(b)

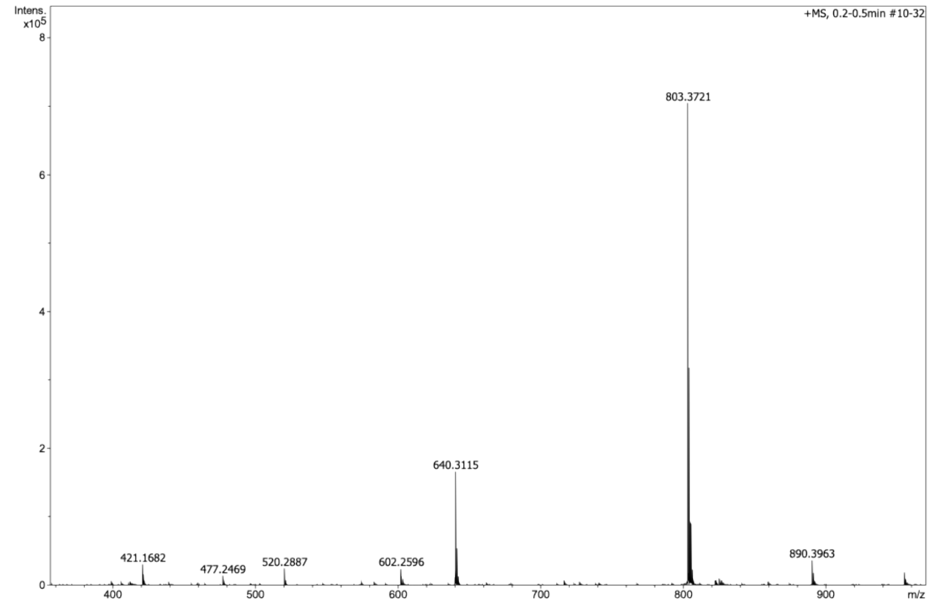

Figure S30. (a) Analytical HPLC profiles of crude dermorphin obtained by ASPPS employing nanoassemblies of Fmoc-amino acids with TATU and DIEA. Elution was carried out over 40 min at a flow rate of 1 mL/min with a linear gradient from 90:10 to 50:50 mixture of 0.1% aqueous TFA and 0.1% TFA in acetonitrile. A dominant peak was observed at a retention time of 18.6 min with a calculated purity of 86.8%; (b) ESI-MS (TOF) spectra. The observed mass was m/z 803.3705 ($[M+H]^+$), which corresponds to $C_{40}H_{51}N_8O_{10}$ (calculated: 803.3728).

5-5. β-Endorphin

(a)

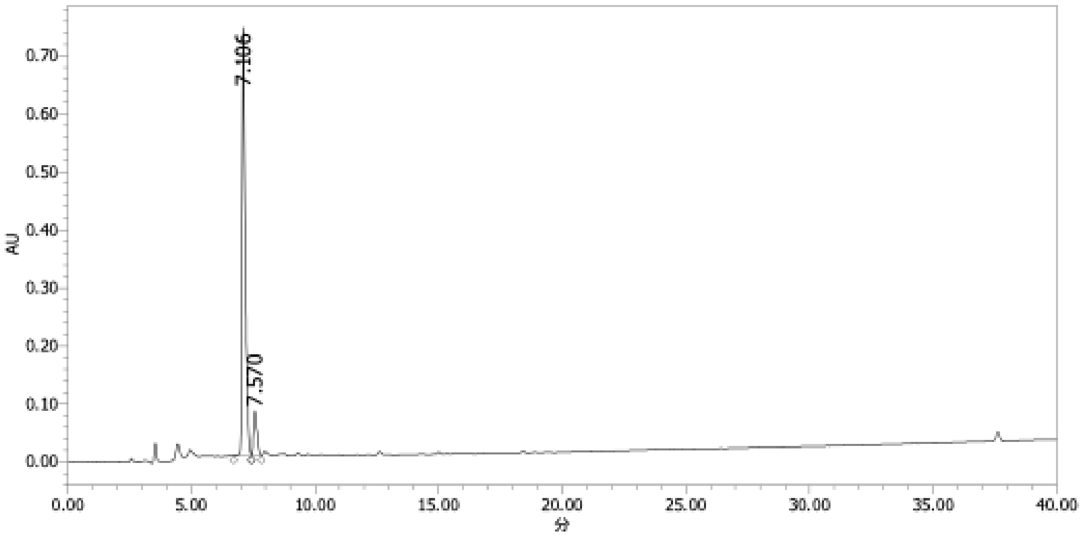

(b)

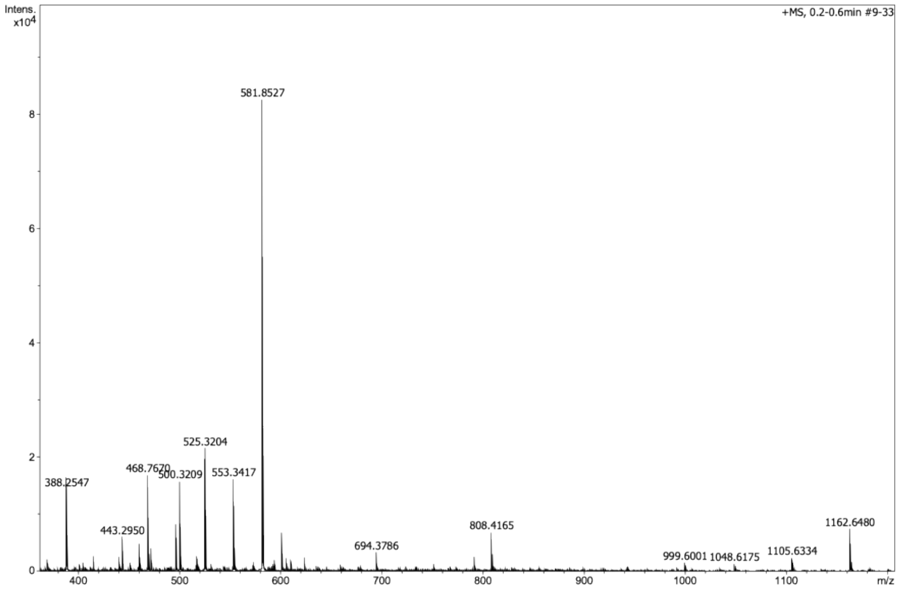

Figure S31. (a) Analytical HPLC profiles of crude β-Endorphin (22-31) obtained by ASPPS employing nanoassemblies of Fmoc-amino acids with TBTU and DIEA. Elution was carried out over 40 min at a flow rate of 1 mL/min with a linear gradient from 90:10 to 50:50 mixture of 0.1% aqueous TFA and 0.1% TFA in acetonitrile. A dominant peak was observed at a retention time of 7.1 min with a calculated purity of 83.6%; (b) ESI-MS (TOF) spectra. The observed monoisotopic mass was m/z 1162.6480 ($[M+H]^+$), which corresponds to $C_{53}H_{92}N_{15}O_{14}$ (calculated: 1162.6948). In addition, a doubly charged species was detected at m/z 581.857 ($[M+2H]^{2+}$).

(a)

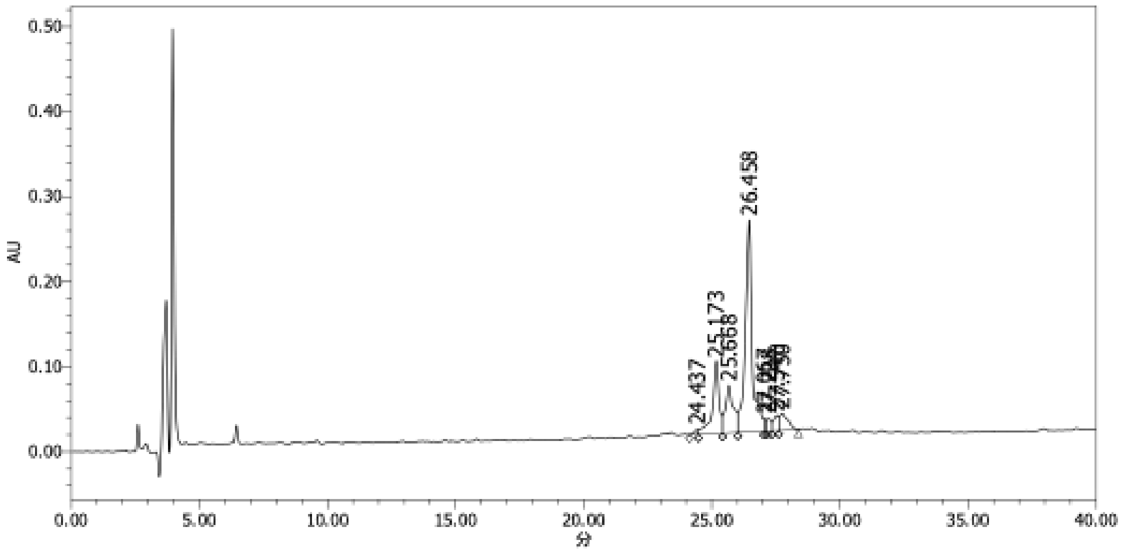

(b)

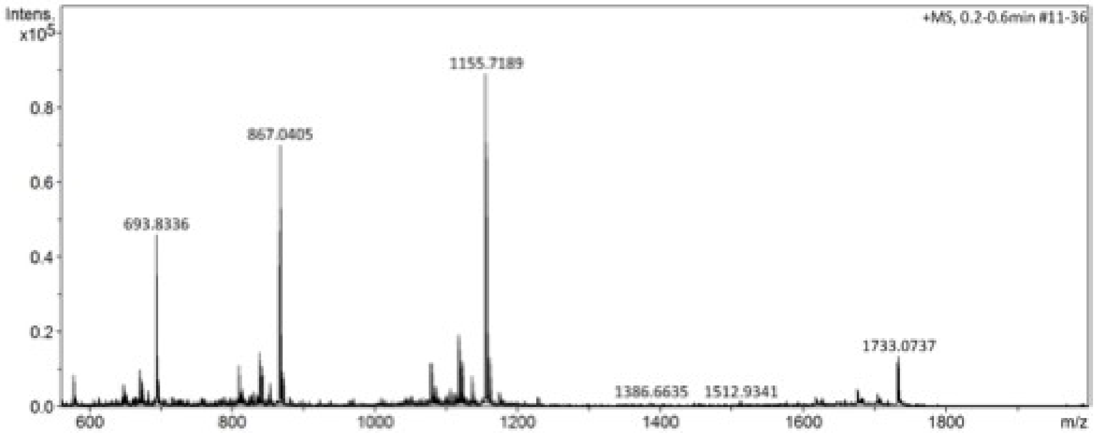

(c)

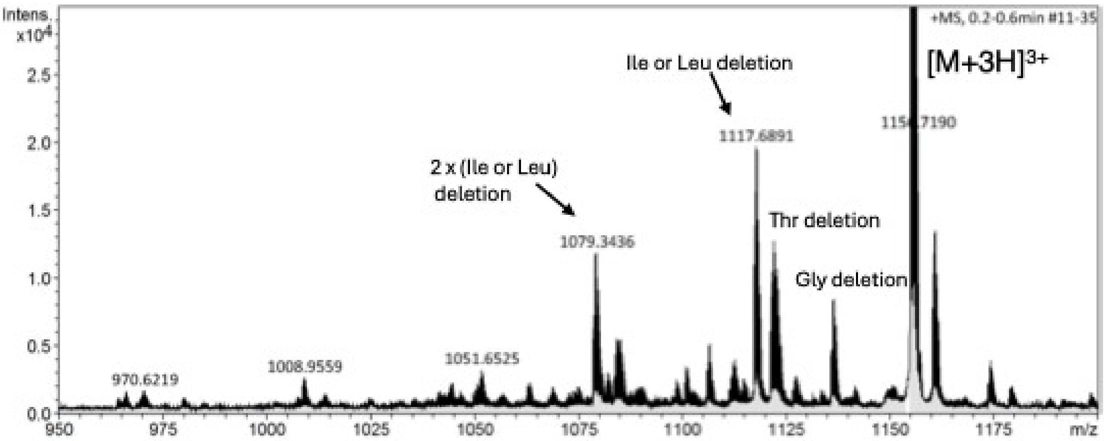

Figure S32. (a) Analytical HPLC profiles of crude β-Endorphin (1-31) obtained by ASPPS employing

nanoassemblies of Fmoc-amino acids with TBTU and DIEA. Elution was carried out over 40 min at a flow rate of 1 mL/min with a linear gradient from 90:10 to 50:50 mixture of 0.1% aqueous TFA and 0.1% TFA in acetonitrile. A dominant peak was observed at a retention time of 26.5 min with a calculated purity of 55.6%; (b) ESI-MS (TOF) spectra. The observed doubly charged species was m/z 1733.0737 ($[M+2H]^{2+}$), which corresponds to $C_{158}H_{253}N_{40}O_{45}S$ (calculated monoisotopic mass: 3463.8537). In addition, a triply charged species was detected at m/z 1155.7189 ($[M+3H]^{3+}$), consistent with the same molecular formula; (c) Expanded view of the MS spectrum supporting the assignment of deletion-related peaks.

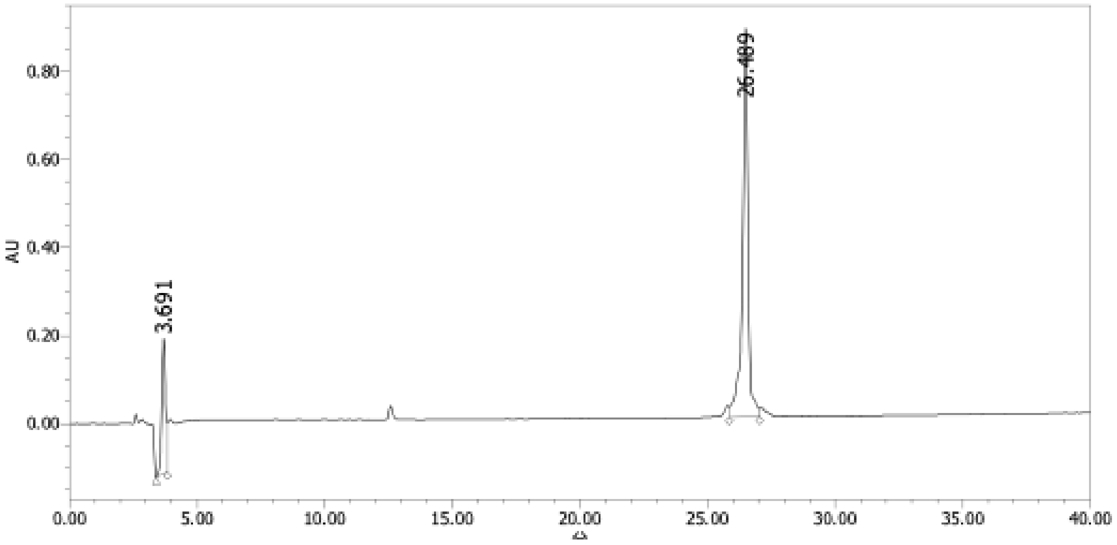

Figure S33. Analytical HPLC profiles of purified β-Endorphin (1-31) obtained by ASPPS employing nanoassemblies of Fmoc-amino acids with TBTU and DIEA. Elution was carried out over 40 min at a flow rate of 1 mL/min with a linear gradient from 90:10 to 50:50 mixture of 0.1% aqueous TFA and 0.1% TFA in acetonitrile. The single peak was observed at a retention time of 26.5 min.

(a)

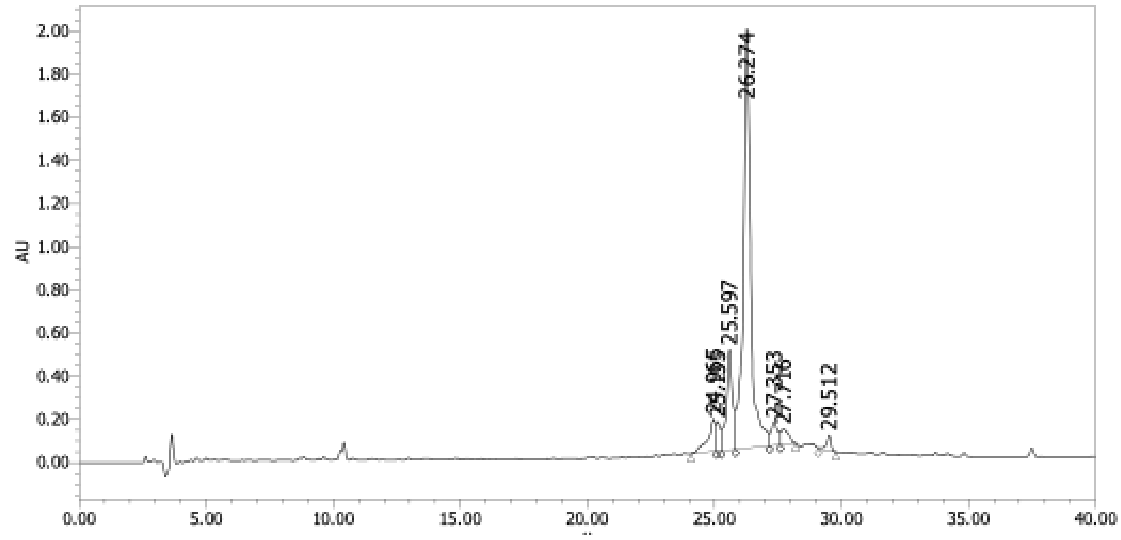

(b)

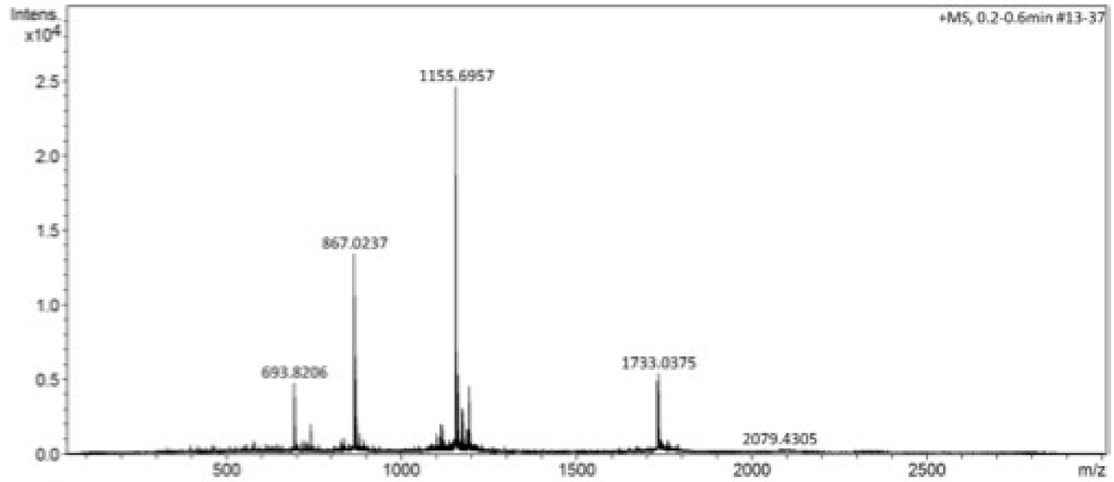

Figure S34. Analytical HPLC profiles of crude β-Endorphin (1-31) obtained by conventional SPPS employing Fmoc strategy in DMF. Elution was carried out over 40 min at a flow rate of 1 mL/min with a linear gradient from 90:10 to 50:50 mixture of 0.1% aqueous TFA and 0.1% TFA in acetonitrile. A dominant peak was observed at a retention time of 26.3 min with a calculated purity of 70.2%; (b) ESI-MS (TOF) spectra. The observed doubly charged species was m/z 1733.0735 ($[M+2H]^{2+}$), which corresponds to $C_{158}H_{253}N_{40}O_{45}S$ (calculated monoisotopic mass: 3463.8537). In addition, a triply charged species was detected at m/z 1155.6957 ($[M+3H]^{3+}$), consistent with the same molecular formula.

6. References

1. ISO 22412:2017, Particle size analysis — Dynamic light scattering (DLS), International Organization for Standardization, Geneva, 2017.
2. B. J. Frisken, *Appl. Opt.*, 2001, **40**, 4087–4091.
3. Malvern Panalytical, “What is the Polydispersity Index (PDI) in Dynamic Light Scattering (DLS)?”, Knowledge Center, 2025.
4. NanoTemper Technologies, “Dynamic light scattering (DLS) — Cumulant Analysis vs. Size Distribution Analysis”, Technical Note.
5. N. Farkas and J. A. Kramar, *J. Nanopart. Res.*, 2021, **23**, 120.
6. A. G. Mailer, P. S. Clegg, P. N. Pusey, *arXiv*, 2015, 1504.06502.
